# Candida auris Cell Wall Mannosylation Contributes to Neutrophil Evasion through Pathways Divergent from Candida albicans and Candida glabrata

**DOI:** 10.1128/mSphere.00406-21

**Published:** 2021-06-23

**Authors:** Mark V. Horton, Chad J. Johnson, Robert Zarnowski, Brody D. Andes, Taylor J. Schoen, John F. Kernien, Douglas Lowman, Michael D. Kruppa, Zuchao Ma, David L. Williams, Anna Huttenlocher, Jeniel E. Nett

**Affiliations:** a Department of Medicine, University of Wisconsin, Madison, Wisconsin, USA; b Department of Medical Microbiology and Immunology, University of Wisconsin, Madison, Wisconsin, USA; c Department of Surgery, Center for Inflammation, Infectious Disease and Immunity, East Tennessee State University, Johnson City, Tennessee, USA; d Department of Biomedical Sciences, Center for Inflammation, Infectious Disease and Immunity, East Tennessee State University, Johnson City, Tennessee, USA; e Department of Pediatrics, University of Wisconsin, Madison, Wisconsin, USA; Duke University Medical Center

**Keywords:** *Candida*, *Candida auris*, mannan, cell wall, neutrophil, immune evasion, innate immunity, glucan masking, Rac2

## Abstract

Candida auris, a recently emergent fungal pathogen, has caused invasive infections in health care settings worldwide. Mortality rates approach 60% and hospital spread poses a public health threat. Compared to other *Candida* spp., C. auris avoids triggering the antifungal activity of neutrophils, innate immune cells that are critical for responding to many invasive fungal infections, including candidiasis. However, the mechanism underpinning this immune evasion has been largely unknown. Here, we show that C. auris cell wall mannosylation contributes to the evasion of neutrophils *ex vivo* and in a zebrafish infection model. Genetic disruption of mannosylation pathways (*PMR1* and *VAN1*) diminishes the outer cell wall mannan, unmasks immunostimulatory components, and promotes neutrophil engagement, phagocytosis, and killing. Upon examination of these pathways in other *Candida* spp. (Candida albicans and Candida glabrata), we did not find an impact on neutrophil interactions. These studies show how C. auris mannosylation contributes to neutrophil evasion though pathways distinct from other common *Candida* spp. The findings shed light on innate immune evasion for this emerging pathogen.

**IMPORTANCE** The emerging fungal pathogen Candida auris presents a global public health threat. Therapeutic options are often limited for this frequently drug-resistant pathogen, and mortality rates for invasive disease are high. Previous study has demonstrated that neutrophils, leukocytes critical for the antifungal host defense, do not efficiently recognize and kill C. auris. Here, we show how the outer cell wall of C. auris promotes immune evasion. Disruption of this mannan polysaccharide layer renders C. auris susceptible to neutrophil killing *ex vivo* and in a zebrafish model of invasive candidiasis. The role of these mannosylation pathways for neutrophil evasion appears divergent from other common *Candida* species.

## INTRODUCTION

Since its description in 2009, Candida auris has continued to spread in health care settings, producing outbreaks of invasive candidiasis globally (1–4). It is the only fungal pathogen categorized as a global public health threat, based on its ability to spread efficiently person-to-person and cause fatal disease (3–6). In an area where it first emerged, the frequency of C. auris bloodstream infections has surpassed that of Candida albicans (7). C. auris frequently exhibits antifungal resistance, with nearly half of the isolates displaying resistance to two or more antifungal drug classes (6). However, even with appropriate antifungal treatment, mortality rates are high (6). In light of the limited treatment options for invasive C. auris infection, and the associated mortality, further understanding of C. auris pathogenicity is of great interest.

Neutrophils are the predominant innate immune cells for control of a variety of fungal pathogens, including *Candida* spp. (8, 9). Neutrophils kill fungi through phagocytosis or the release of neutrophil extracellular traps (NETs) (10–12). Phagocytosis appears to be effective against single-cell yeast forms, while NETs demonstrate activity against larger hyphal forms (13). However, in response to C. auris, neutrophils fail to efficiently engage in phagocytosis or release NETs (14). Here, we explore a role for cell wall mannosylation in neutrophil evasion for C. auris and find that disruption of this outer polysaccharide layer promotes phagocytosis and fungal killing. Strikingly, genetic disruption of the same mannosylation pathways in other *Candida* spp. does not influence phagocytosis, suggesting a divergent role for these fungal pathways during C. auris*-*host interactions.

## RESULTS

### Multiple strains of C. auris exhibit evasion of neutrophil phagocytosis.

**FIG 1:**
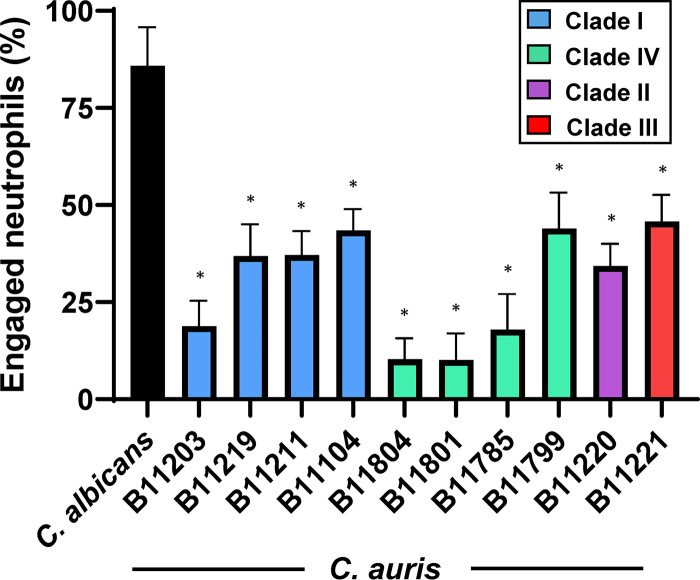
C. auris evasion of neutrophil phagocytosis is demonstrated across multiple strains. Human neutrophils from healthy donors were incubated for 1 h with C. albicans SN250 or C. auris strains labeled with calcofluor white and subsequently imaged via fluorescence microscopy. The numbers of neutrophils engulfing fungal cells were counted and the percentages of total engaged neutrophils were calculated. High power fields (*n* = 8 to 10) were examined with neutrophils from at least two donors. *, *P* < 0.05 by one-way ANOVA with Holm-Sidak multiple comparisons to C. albicans.

Our previous work demonstrated that C. auris (strain B11203, a clinical isolate originating from India) avoided triggering antifungal neutrophil responses when compared to C. albicans (14). To determine if evasion of neutrophil response is a conserved trait across a variety of C. auris strains, we measured neutrophil phagocytosis for a panel of ten C. auris isolates collected by the Centers for Disease Control and Prevention (6). As previously described, we found C. albicans to be highly engulfed by neutrophils, while C. auris B11203 exhibited a greatly reduced susceptibility to neutrophil phagocytosis, with only 15 to 20% of neutrophils engaged (Fig. 1) (14). Notably, all the C. auris strains displayed significantly lower susceptibility to neutrophil phagocytosis compared to C. albicans. Furthermore, three strains isolated from Colombia (B11804, B11801, and B11785) demonstrated a particularly low susceptibility to neutrophil engulfment (10 to 20%), comparable or lower than that observed for strain B11203. Taken together, these results demonstrated that diverse C. auris strains from a variety of clades exhibit significantly decreased susceptibility to neutrophil phagocytosis compared to C. albicans. Based on our prior study, we elected to move forward with strain B11203 to characterize the mechanism of neutrophil evasion in this emergent pathogen through investigation of the yeast cell wall.

### Genetic disruption of C. auris mannosylation enhances phagocytosis.

**FIG 2:**
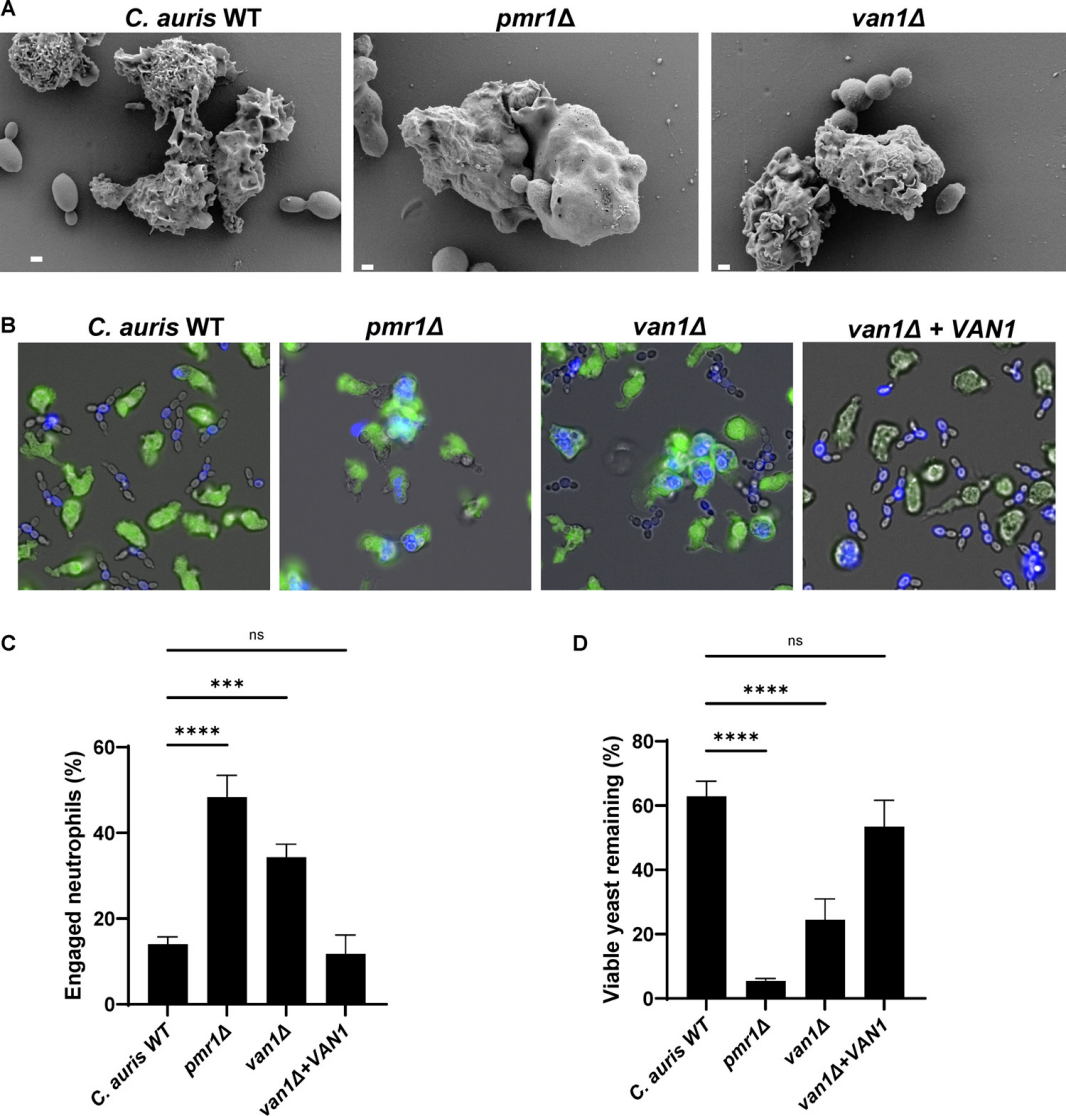
C. auris mannosylation pathway mutants are susceptible to neutrophil attack. (A) C. auris strains were incubated with human neutrophils for 1 h and were subsequently imaged via scanning electron microscopy. Images are 10,000× magnification, measurement bars represent 1 μm. (B and C) Human neutrophils were labeled with calcein-AM (green) and cocultured with individual C. auris strains labeled with calcofluor white (blue) for 1 h and imaged via fluorescence microscopy (B). The numbers of neutrophils engulfing fungal cells were counted and the percentages of total engaged neutrophils were calculated (C); *n* ≥ 3, mean with standard error of the mean (SEM) shown. (D) Individual C. auris strains were cultured with human neutrophils for 4 h and viable burden was estimated by PrestoBlue metabolic activity following neutrophil lysis; *n* = 3, mean with standard deviation shown. *, *P* < 0.05; **, *P* < 0.01; ***, *P* < 0.001; ****, *P* < 0.0001; ns, not significant by one-way ANOVA with Holm-Sidak multiple comparisons to C. auris WT.

To explore the influence of cell wall mannosylation on neutrophil evasion for C. auris, we elected to construct mutants, targeting pathways based on their functions described for C. albicans or S. cerevisiae (15–17). We first targeted *PMR1* because of its broad role in mannosylation (18). In C. albicans, *PMR1* disruption impairs both *O*- and *N-*mannosylation, resulting in truncated cell wall structures (18). We identified a homolog using BLAST searches through available sequence data for C. auris. For disruption, we replaced *PMR1* with a selectable marker (*NAT1*) (19). By scanning electron microscopy and fluorescence microscopy, we observed a heightened capacity for human neutrophils to engulf this mutant strain (Fig. 2A and B). Compared to the parent strain, neutrophils engaged the *pmr1*Δ mutant at a greater than 3-fold higher rate (Fig. 2C). Strikingly, this mutant also displayed a high susceptibility to killing by human neutrophils (Fig. 2D).

The cell walls of C. auris, like C. albicans, contain a higher abundance of *N-*linked mannans compared to *O-*linked mannans (20). We hypothesized that highly branched *N-*mannans may contribute to neutrophil evasion for C. auris. As a second method to disrupt *N-*mannosylation, we similarly constructed a *van1*Δ mutant lacking a putative α-1,6 mannosyltransferase required for the elongation of the *N*-mannan backbone (17, 21). Similar to the *pmr1*Δ mutant strain, human neutrophils efficiently phagocytosed the *van1Δ* mutant at a 2- to 3-fold greater rate compared to the parent strain, as visualized by fluorescence microscopy and scanning electron microscopy (Fig. 2A to C). This heightened neutrophil response also led to increased killing of the *van1*Δ strain when compared to the parent strain (Fig. 2D). Complementation of a *VAN1* allele increased *VAN1* expression (Fig. S1 in the supplemental material) and reversed the *van1*Δ phenotypes, with the *van1*Δ + *VAN1* strain displaying neutrophil evasion similar to the parent strain (Fig. 2B to D).

10.1128/mSphere.00406-21.6 FIG S1 C. auris mutants lack expression of targeted genes. Reverse transcriptase PCR (RT-PCR) was performed on isolated RNA from each strain, where the expression of each gene is presented relative to that of *ACT1*. Download FIG S1, TIF file, 1.0 MB. Copyright © 2021 Horton et al. 2021 Horton et al. https://creativecommons.org/licenses/by/4.0/ This content is distributed under the terms of the Creative Commons Attribution 4.0 International license.

We next considered the mechanism of killing of *pmr1*Δ and *van1*Δ mutants. While single-cell yeasts can typically be killed upon completion of phagocytosis, the role of NETs for control of fungal pathogens has been increasingly recognized (10, 11, 13). We did not observe NET-like structures by scanning electron microscopy and did not detect the release of extracellular DNA in response to any of the C. auris strains (Fig. S2A). Neutrophils exposed to the *pmr1*Δ and *van1*Δ mutants generated a slight increase in reactive oxygen species compared to the parent strain (Fig. S2B). We also questioned if the mannan mutant strains might be susceptible to oxidative stress produced by neutrophils and thus measured growth in the presence of hydrogen peroxide. We found similar oxidative stress tolerances among the wild-type and mutant strains (MIC = 16 μM). Similarly, the strains were not more susceptible to the oxidative stressor menadione (MIC = 64 μM). Susceptibilities to all tested stressors are listed in Table S1. Taken together, these results suggest that disruption of C. auris mannosylation pathways results in increased susceptibility to neutrophil phagocytosis and killing.

10.1128/mSphere.00406-21.2 TABLE S1 Minimal inhibitory concentration values of cellular stressors. Download Table S1, DOCX file, 0.02 MB. Copyright © 2021 Horton et al. 2021 Horton et al. https://creativecommons.org/licenses/by/4.0/ This content is distributed under the terms of the Creative Commons Attribution 4.0 International license.

10.1128/mSphere.00406-21.7 FIG S2 Neutrophils do not produce NETs, but do show some increased ROS, in response to *pmr1*Δ and *van1*Δ strains. (A) Human neutrophils and C. auris strains were incubated for 4 h, then Sytox Green was added to measure extracellular DNA by fluorescence (500/528nm) in a microplate reader. Phorbol myristate acetate (PMA) was added to a subset of wells serving as a positive control for NET formation, *n* = 3, mean with SEM are shown. *, *P* < 0.05; ns, not significant by one-way ANOVA with Holm-Sidak multiple comparisons to C. auris WT. (B) C. auris strains were incubated with human neutrophils for 4 h in the presence of CM-H_2_DCFDA to measure intracellular ROS generation by fluorescence (495/527 nm) by microplate reader. PMA was added to a subset of wells serving as a positive control for ROS generation, *n* = 4, mean with SEM shown. Download FIG S2, TIF file, 1.2 MB. Copyright © 2021 Horton et al. 2021 Horton et al. https://creativecommons.org/licenses/by/4.0/ This content is distributed under the terms of the Creative Commons Attribution 4.0 International license.

### Disruption of *PMR1* or *VAN1* alters the C. auris cell wall structure though a decrease in mannan.

**FIG 3:**
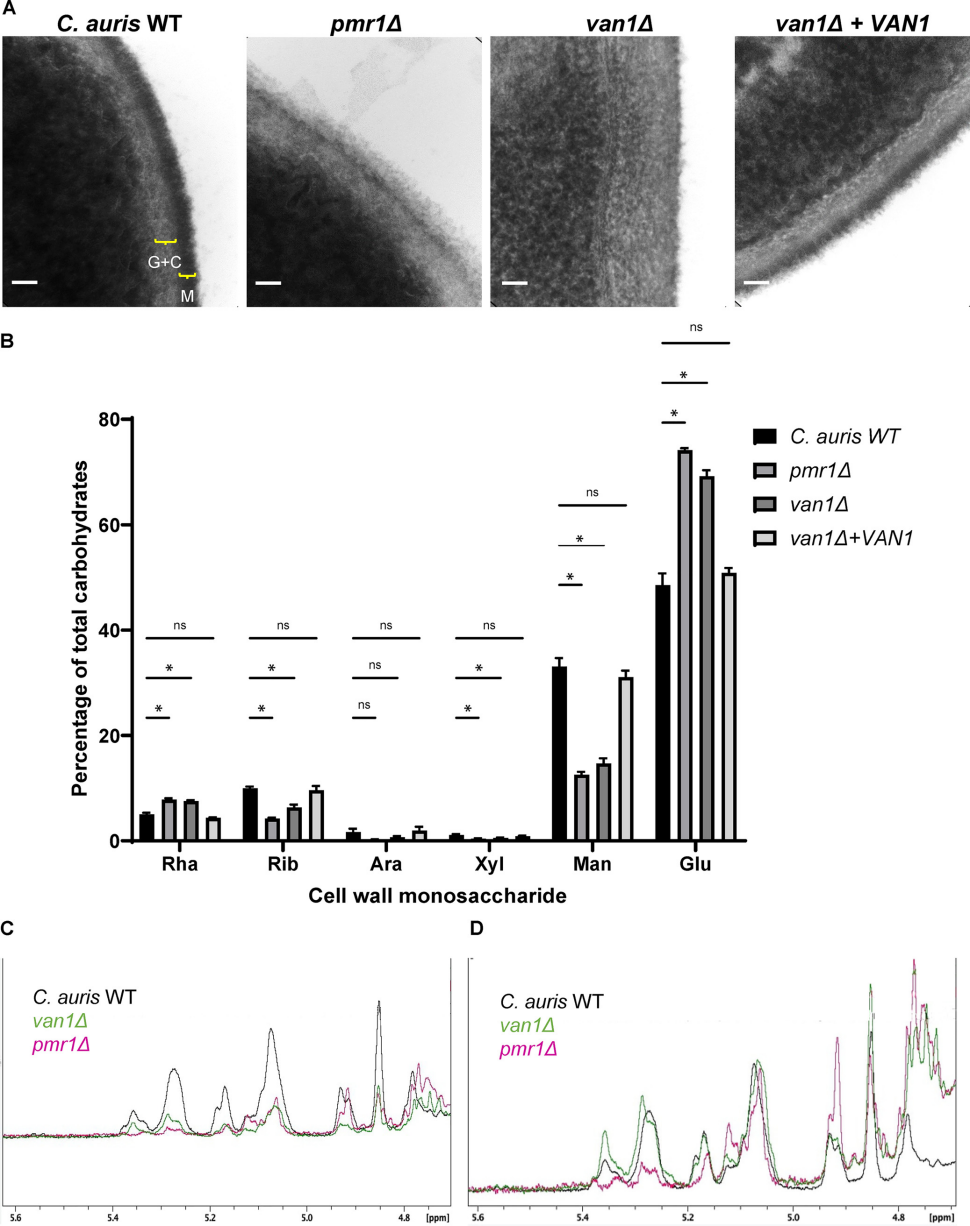
C. auris *pmr1*Δ and *van1*Δ strains display an altered cell wall structure that contains less mannan. (A) C. auris yeast were imaged via transmission electron microscopy at 383,000× magnification, and scale bars represent 50 nm. Brackets denote distinct cell wall layers: G+C, β-glucan and chitin; M, mannan. (B) The monosaccharide compositions of cell walls were measured by gas chromatography, *n* = 5, mean with SEM shown, *, *P* < 0.05; ns, not significant by one-way ANOVA with Holm-Sidak multiple comparisons to C. auris WT. Rha, rhamnose; Rib, ribose; Ara, arabinose; Xyl, xylose; Man, mannose; Glu, glucose. (C and D) The structures of isolated mannans were analyzed by ^1^H NMR and COSY spectra. C shows the ^1^H NMR spectra for each strain following mannan isolation. In panel D, the intensities were adjusted to the resonance assigned to sidechain-linked backbone α1-6-linked mannosyl repeat units (5.07 ppm) to compare mannan structures.

We next aimed to characterize changes in the cell walls associated with disruption of *PMR1* or *VAN1*. We first analyzed the cell walls of the wild-type and mutant strains using transmission electron microscopy. The wild-type strain exhibited a dense outer layer, consistent with a mannan-rich outer cell wall (M) (22). Adjacent to this, we observed a more lucent inner cell wall layer, which has been shown to contain glucan and chitin residues (G+C) (Fig. 3A). In contrast, the outer cell walls of both the *pmr1*Δ and *van1*Δ mutants appeared less dense. The loss of a dense mannan layer is consistent with a decrease in cell wall mannan (23). In addition, these mutant strains had increased electron lucent layers, suggesting a compensatory increase in glucan and/or chitin (Fig. 3A and Fig. S3).

10.1128/mSphere.00406-21.8 FIG S3 C. auris mutants have altered lengths of cell wall layers. Lengths of cell wall layers from transmission electron microscopy (TEM) images were manually measured using Fiji. Eight different TEM images were utilized for each strain, with three separate length measurements per image, mean with SEM shown; *, *P* < 0.05; ****, *P* < 0.0001; ns, not significant by one-way ANOVA with Holm-Sidak multiple comparisons to C. auris WT. Download FIG S3, TIF file, 1.1 MB. Copyright © 2021 Horton et al. 2021 Horton et al. https://creativecommons.org/licenses/by/4.0/ This content is distributed under the terms of the Creative Commons Attribution 4.0 International license.

To quantify cell wall mannan, we performed monosaccharide analysis of isolated cell walls (Fig. 3B). For the wild-type strain, we found mannose, the building block of mannan, to comprise nearly 40% of the total carbohydrate. In comparison, the *pmr1*Δ and *van1*Δ cell walls contained less than 20% mannose. The observed decrease in mannose was accompanied by an increase in glucose, suggesting that glucans may comprise a greater percentage of the cell wall for these mutant strains. Complementation of the *van1*Δ mutant restored both the cell wall structure and mannan composition (Fig. 3A and B).

Fungal mannans contain a variety of linkages (24, 25). To determine the structural differences associated with disruption of *PMR1* and *VAN1*, we utilized 1D ^1^H and 2D COSY NMR (Table 1 and Fig. 3C and D). As expected from monosaccharide analysis, we observed considerably less mannan for the *pmr1*Δ and *van1*Δ strains based on their lower amplitude in many of the spectral regions (Fig. 3C). In light of this, we also compared the structural motifs after adjusting the intensity of the resonance for the anomeric protons of the α1→6-linked mannosyl repeat units (5.07 ppm) in the backbone chain, so that the resonance intensities would be similar for each strain (Fig. 3D). Structural motifs were determined by analyzing the chemical shifts of the anomeric proton (H1) and its nearest neighbor (H2) in the mannosyl repeat unit observed in the H1-H2 crosspeaks of the 2D COSY spectrum.

**TABLE 1:** Mannan structural motif assignments for wild-type C. auris

H1	H2	Structural motif
5.551	-^*a*^	Manβ1-2(Manβ1-2)_n_Manα1-PO_4_ or Manβ1-2Manα1-PO_4_
5.3764	4.1064	α1-2Manα1-3Manα1-2
5.2845	4.114	Manα1-2Manα1-2
5.269	4.134	-6(Manα1-2)Manα1-6(Manα1-2Manα1-2)Manα1-
5.255	4.133	α1-2Manα1-2
5.168	4.263	Manβ1-2Manα1-2
5.119	4.036	α1-6(-2)Manα1-6(Manα1-2)Manα1-6(-2)Manα1-6
5.092	4.017	-6(Manα1-2)Manα1-6(Manα1-2Manα1-2)Manα1-
5.0766	4.0714	α1-6(-2)Manα1-6(α1-2Manα1-2)Manα1-6(-2)Manα1-6
5.0577	4.0747	-6(Manα1-2)Manα1-6(Manα1-2Manα1-2)Manα1-
5.052	4.209	α1-3Manα1-2
5.035	4.249	α1-3Manα1-2
4.929	4.012	Manα1-6
4.91	4.006	Manα1-6(Manα1-6)_n_Manα1-6
4.853	4.268	Manβ1-2Manβ1-2Manα1-2
4.845	4.156	Manβ1-2Manβ1-2Manβ1-2Manα1-2
4.782	4.058	Manβ1-2Manα1-2

a Not observed in the COSY spectrum.

Overall, the *van1*Δ mutant mannan structure was quite similar to the wild type, but the *pmr1*Δ mutant mannan showed differences in structural motifs (Fig. 3D and Table 1). The levels of acid-labile side chains containing two (Manβ1-2Manα1-PO_4_) and/or more repeat units (Manβ1-[2Manβ1-]_n_2Manα1-PO_4_) were comparable between the wild type and *van1*Δ mutant and present at very low levels in the spectra (5.55 ppm). These sidechains were not observed in the *pmr1*Δ mutant. We did not find evidence for Manα1-PO_4_ acid-labile sidechains in the spectra for any of the strains. Acid-stable sidechains containing (1-3)-linked mannosyl repeat units (-2Manα1-3Manα1-2Manα1-) at 5.36 ppm were approximately twice as abundant in the *van1*Δ mutant compared to the wild type and were not observed in the *pmr1*Δ mutant. The levels of -2Manα1- repeat units (5.284 and 5.255 ppm) in the acid-stable sidechains were comparable in the *van1*Δ mutant and the wild type, while again being much lower in the *pmr1*Δ mutant. The levels of Manβ1-2Manα1-2 sidechain terminating repeat units (5.168 and 4.853 ppm) in the acid-stable sidechains were comparable in the *van1*Δ mutant and the wild type and slightly lower in the *pmr1*Δ mutant. The levels of Manα1-2 single repeat unit sidechains attached to the backbone (5.123 ppm) with longer (1-2)-linked sidechains surrounding it were comparable in the wild type and *van1*Δ mutant, but higher in the *pmr1*Δ strain. Lastly, the levels of unsubstituted (1-6)-linked backbone repeat units (4.93 and 4.91 ppm) were comparable in the wild type and *van1*Δ strain but higher in the mutant *pmr1*Δ. Overall, these results indicate reduced mannan content in the *van1*Δ and *pmr1*Δ strains. Furthermore, the structure of remaining mannan in *van1*Δ is similar to wild type, but the *pmr1*Δ displayed some mannan structural motifs that were different from those observed in the wild type. This suggested to us that the observed neutrophil evasion phenotype was due to an overall quantitative decrease in mannans.

### C. auris *pmr1*Δ and *van1*Δ strains display increased pathogen-associated molecular patterns.

We next questioned how C. auris mannosylation pathways might be influencing phagocytosis by neutrophils. Because the *van1*Δ mannan structure was comparable to wild-type mannan, it appeared less likely that neutrophils were engaging *van1*Δ through interaction with an altered mannan structure. Rather, since both the *van1*Δ and *pmr1*Δ cell walls displayed an overall decrease in mannan content (Fig. 3B and C), we theorized that the overall lack of mannosylation was likely to be contributing to the increased phagocytosis observed for these mutants. We reasoned that disruption of the mannoprotein layer could unmask immunostimulatory cell wall pathogen-associated molecular patterns (PAMPs), thus promoting neutrophil engagement (26–29).

**FIG 4:**
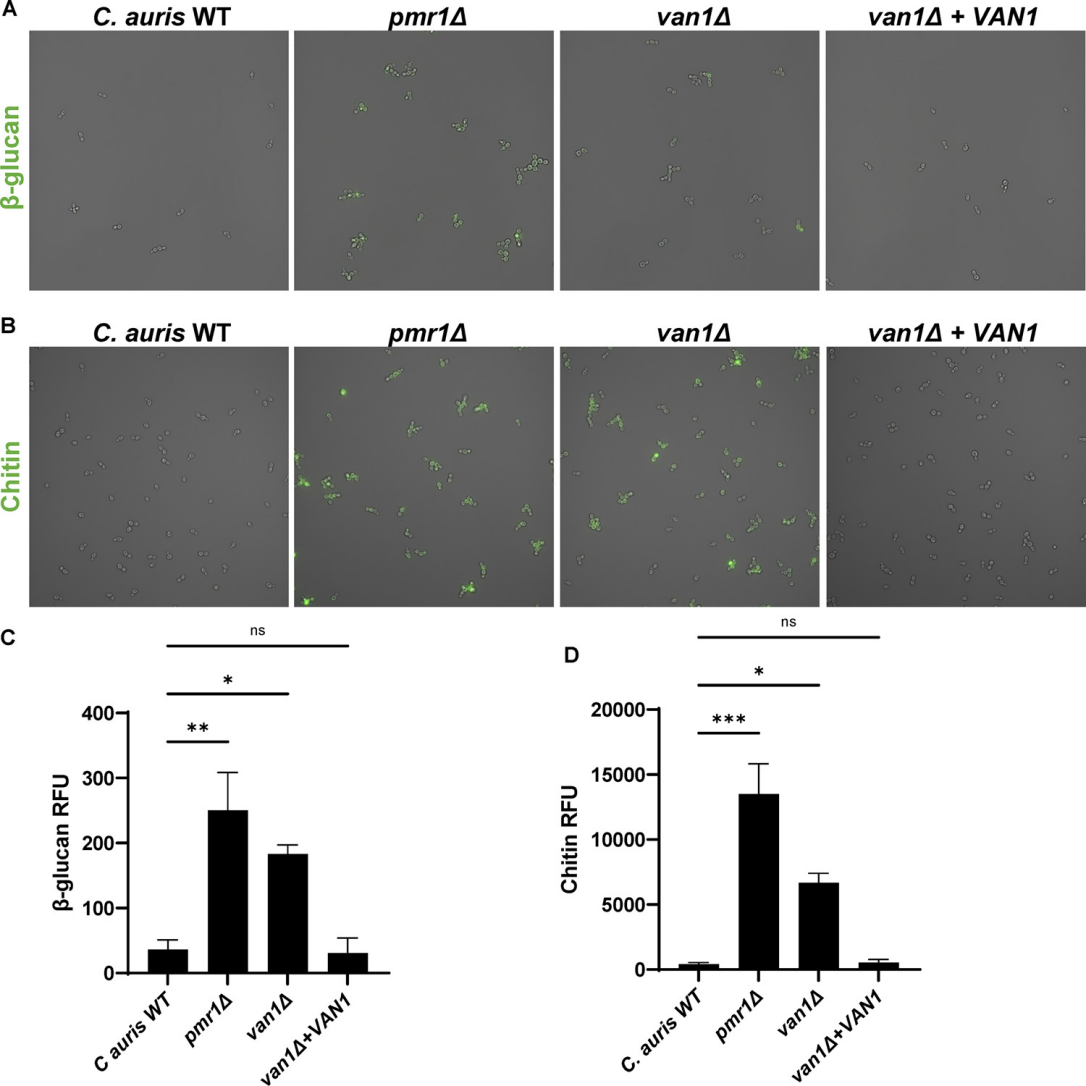
C. auris *pmr1*Δ and *van1*Δ strains display increased cell surface PAMPs. (A) Cell surface β-glucan was labeled using Fc:dectin-1 protein with Alexa Fluor 488-conjugated anti-human IgG Fc antibody and imaged by fluorescence microscopy. (B) Cell surface chitin was labeled with wheat germ agglutinin conjugated to fluorescein isothiocyanate (WGA-FITC) and assessed by fluorescence microscopy. (C and D) Total surface β-glucan and chitin were quantified by plate reader measurements of fluorescence, *n* = 3 mean with SEM shown, *, *P* < 0.05; **, *P* < 0.01; ***, *P* < 0.001 by one-way ANOVA with Holm-Sidak multiple comparisons to C. auris WT; ns, not significant.

To analyze the cell-surface display of PAMPs in the setting of mannosylation disruption, we utilized immunofluorescence, examining β-glucan and chitin with recombinant dectin-1 and wheat germ agglutinin, respectively (30, 31). The wild-type C. auris strain exhibited very little cell surface β-glucan or chitin (Fig. 4). In contrast, *pmr1*Δ and *van1*Δ strains displayed both β-glucan and chitin, with quantification of fluorescence revealing 5 to 100× greater PAMP exposure. Complementation of *VAN1* in the *van1*Δ strain reversed PAMP exposure. Taken together, these findings indicate increased exposure of immunostimulatory PAMPs accompanies the decreased cell wall mannan content of *pmr1*Δ and *van1*Δ strains.

### C. auris *pmr1*Δ and *van1*Δ strains exhibit increased neutrophil recruitment to the zebrafish hindbrain and fail to grow to high burdens.

**FIG 5:**
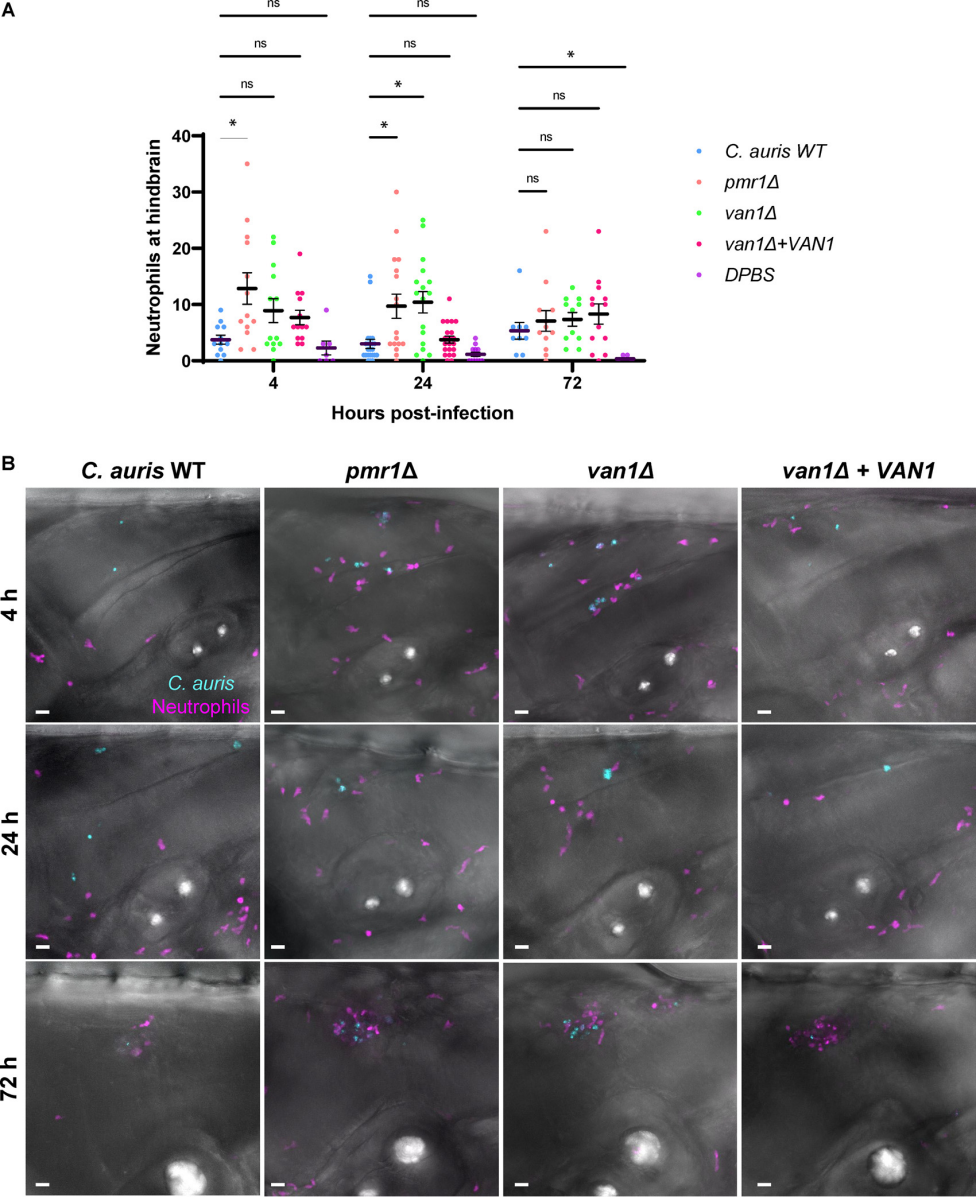
C. auris mannan mutants stimulate increased neutrophil recruitment in the larval zebrafish hindbrain. C. auris strains were injected into the hindbrains of larvae from a cross between the *Tg*(*lyzC:RFP*) and *Tg*(*mpeg:GFP*) lines at 2 days postfertilization. Fluorescence microscopy was utilized to measure recruitment of neutrophils to the hindbrain at 4, 24, and 72 h postejection. (A) At each time point, fluorescent neutrophils were manually enumerated from maximum intensity projections from z-stacks; *n* = 9 to 23, experiments were performed in three replicates; the mean with SEM are shown; *, *P < *0.05; ns, not significant by Brown-Forsythe and Welch ANOVA with Dunnett’s T3 multiple comparisons to C. auris WT. (B) Representative fluorescence microscopy images of neutrophil recruitment to zebrafish hindbrain are shown (magenta = neutrophils, cyan = C. auris cells). Scale bar = 20 μm.

To examine the influence of C. auris cell wall mannosylation on neutrophil activity *in vivo*, we utilized a zebrafish larvae model (14, 32–34). The translucent larvae are ideal for the tracking and imaging of leukocytes. We performed hindbrain injections of C. auris in double-transgenic zebrafish larvae *Tg*(*mpeg1:EGFP*)×*Tg*(*lyzc:tagRFP*), with fluorescently labeled macrophages and neutrophils, respectively (35, 36). Over the course of the first 24 h after injection, very few neutrophils recruited to the hindbrain of zebrafish injected with the C. auris wild-type strain (Fig. 5A and B). The numbers of neutrophils in the hindbrain were similar to saline-only injection controls. This lack of neutrophil recruitment and engagement is consistent with human neutrophil experiments and a prior zebrafish study (Fig. 1) (14). In contrast, zebrafish neutrophils recruited to the *pmr1*Δ and *van1*Δ mutant strains at an approximately 3- to 5-fold greater rate over this initial time period. Fluorescence imaging revealed the neutrophil phagocytosis of these mutant strains (Fig. 5B). By 72 h, neutrophil recruitment appeared similar among the strains. We also considered that C. auris mannosylation might affect macrophage recruitment. However, we observed similar macrophage recruitment to wild-type and mutant strains (Fig. S4).

10.1128/mSphere.00406-21.9 FIG S4 Macrophages similarly migrate to C. auris *pmr1*Δ and *van1*Δ in a zebrafish infection model. (A) Fluorescence microscopy was utilized with larvae from the *Tg*(*lyzC:RFP*) line crossed with the *Tg*(*mpeg:GFP*) line to measure recruitment of macrophages to zebrafish larvae hindbrains at 4 h, 24 h, and 72 h. At each time point, fluorescent macrophages were manually enumerated from maximum intensity projections from z-stacks. At least 9 fish were used for each individual strain of C. auris, experiments were performed in three replicates, mean with SEM are shown; *P < 0.05; ns, not significant by Brown-Forsythe and Welch ANOVA with Dunnett’s T3 multiple comparisons to C. auris WT. Download FIG S4, TIF file, 1.1 MB. Copyright © 2021 Horton et al. 2021 Horton et al. https://creativecommons.org/licenses/by/4.0/ This content is distributed under the terms of the Creative Commons Attribution 4.0 International license.

**FIG 6:**
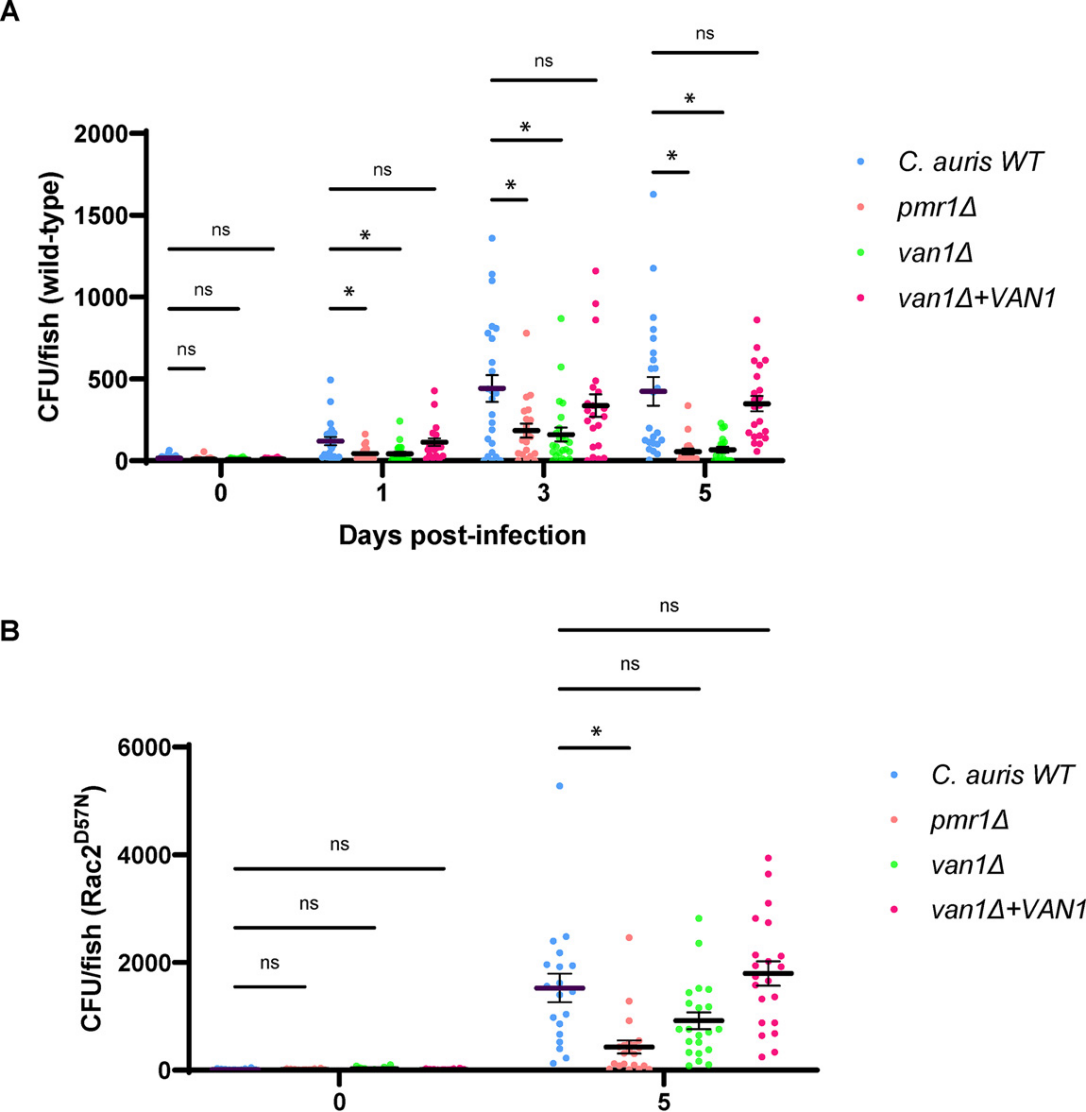
C. auris *pmr1*Δ and *van1*Δ strains grow to lower burdens in the larval zebrafish hindbrain in the presence of neutrophils. Wild-type (A) or transgenic zebrafish expressing a dominant Rac2^D57N^ mutation in neutrophils (B) were inoculated with C. auris by hindbrain injection. Fungal burden was quantified at 0, 1, 3, and/or 5 days postinfection by homogenizing whole larvae and plating for CFU. *n* = 20 to 24; experiments were performed in three replicates; mean with SEM are shown; *, *P < *0.05; ns, not significant by Brown-Forsythe and Welch ANOVA with Dunnett’s T3 multiple comparisons to C. auris WT.

We next analyzed the impact of C. auris mannosylation on virulence in the zebrafish infection model. Following hindbrain injection, we found the wild-type C. auris to propagate over the 5-day course of the experiments, ultimately reaching a burden close to 500 CFU/fish (Fig. 6A). In contrast, the burdens for *pmr1*Δ and *van1*Δ mutants were 2- to 3-fold lower over the course of the first 3 days. By 5 days, many of the fish injected with these mutant strains had completely cleared the infection. Complementation of *VAN1* in the *van1*Δ mutant reversed the virulence defect. We considered the possibility that the mutant strains may display a growth defect in the conditions of the zebrafish infection model, but did not observe differences in growth at 29°C among the strains (Fig. S5). The low burdens of the mutant strains *in vivo* are consistent with the fungal killing through increased neutrophil recruitment and phagocytosis (Fig. 5A and B).

10.1128/mSphere.00406-21.10 FIG S5 C. auris *pmr1*Δ and *van1*Δ strains have similar growth curves compared to C. auris WT. C. auris strains in RPMI supplemented with 2% fetal bovine serum (FBS) were added in triplicate to a clear 96-well microtiter plate and absorbance at 600 nm was measured every hour for 18 h at 29°C (*n* = 3). Download FIG S5, TIF file, 1.1 MB. Copyright © 2021 Horton et al. 2021 Horton et al. https://creativecommons.org/licenses/by/4.0/ This content is distributed under the terms of the Creative Commons Attribution 4.0 International license.

To examine the specific role of neutrophils in elimination of the C. auris strains, we utilized a transgenic zebrafish line expressing a dominant Rac2^D57N^ mutation specifically in neutrophils (Tg[mpx:mCherry-2A-Rac2D57N]) (D57N) (37). These fish have a mutation that mimics a form of leukocyte adhesion deficiency in humans in which neutrophils lack the capacity to migrate from circulation or recruit to infection. In this hindbrain infection model without functional neutrophils, we found the *pmr1*Δ and *van1*Δ mutants to grow to over 7.5-fold and 13.5-fold greater burdens by day 5, respectively, compared to infection of wild-type fish (Fig. 6A and B). This supports the importance of neutrophils for killing these mutant strains in wild-type fish. The finding that the *van1*Δ mutant grew to a burden similar to wild-type C. auris in the fish lacking functional neutrophils shows that the mutant strain does not have a general *in vivo* growth defect and that the neutrophils are the major contributor to clearance of *van1*Δ in wild-type fish.

### Cell wall mannosylation pathway genes function distinctly for neutrophil evasion in C. auris compared to other *Candida* species.

**FIG 7:**
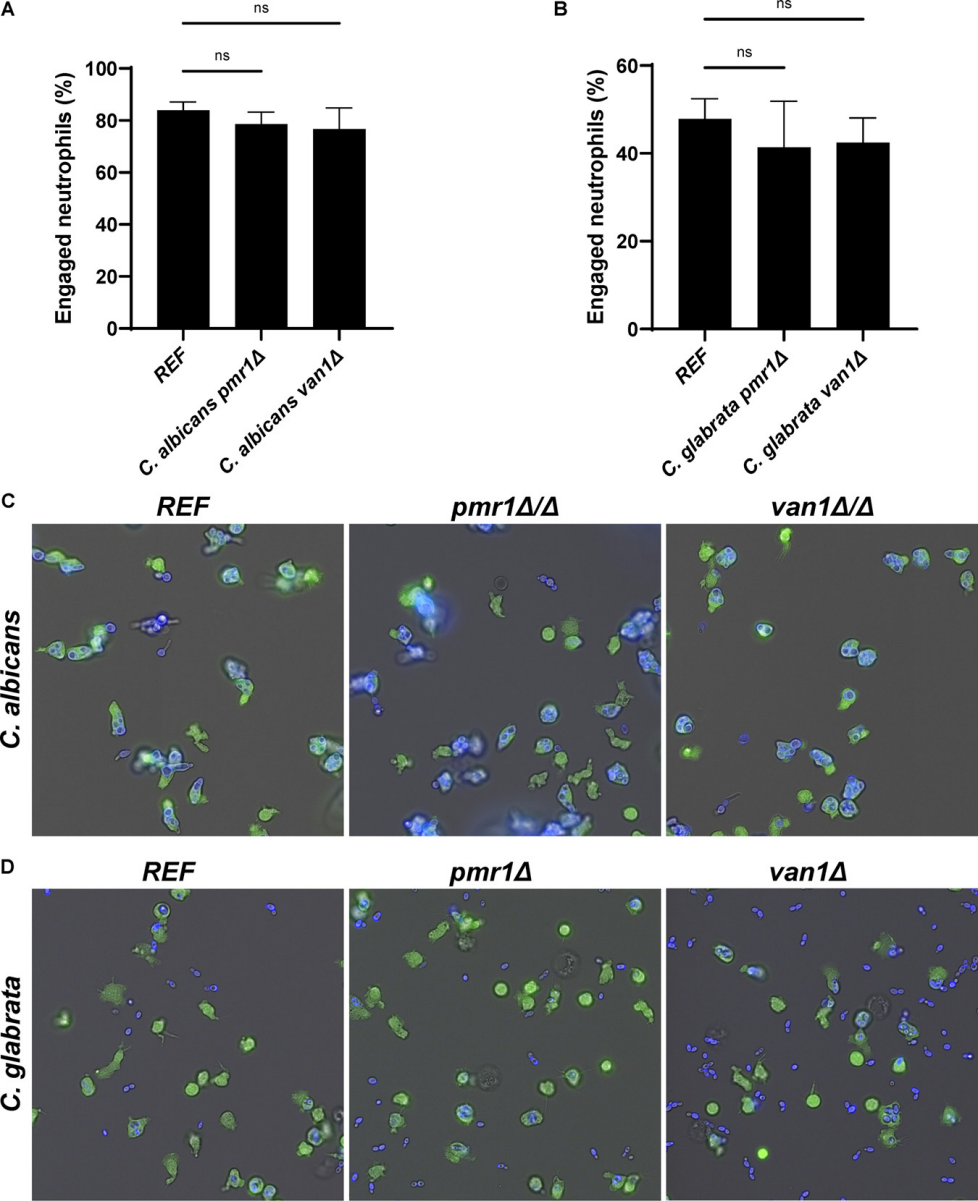
*PMR1* and *VAN1* do not influence neutrophil engagement of Candida albicans or Candida glabrata. Human neutrophils were labeled with calcein-AM and cocultured with calcofluor white-labeled C. albicans (A) or C. glabrata (B) for 1 h and imaged via fluorescence microscopy. The number of neutrophils engulfing yeast cells was counted and the percentage of total engaged neutrophils was calculated, *n* = 3, mean with SEM are shown; analyzed by one-way ANOVA with Holm-Sidak multiple comparisons to REF, ns = not significant. Representative fluorescence microscopy images are shown for C. albicans (C) and C. glabrata (D).

We next questioned if the cell wall mannosylation genes *PMR1* and *VAN1* influence neutrophil responses for other *Candida* species. We selected C. albicans and C. glabrata for comparison. C. albicans is a polymorphic species with the capacity for filamentation, while C. glabrata proliferates only as a yeast, with a small size similar to C. auris. Neutrophils exhibited a propensity to engage C. albicans beyond that observed for C. auris, similar to prior study (Fig. 1 and 7A) (14). Homozygous disruption of either *PMR1* or *VAN1* did not influence neutrophil engagement. Similarly, no differences in neutrophil-*Candida* interactions were observed for C. glabrata mutants lacking *PMR1* or *VAN1* compared to the reference strain (Fig. 7B). These results suggest that disruption of mannosylation pathway genes in these *Candida* species does not result in increased susceptibility to neutrophil phagocytosis. The findings support unique roles for C. auris *PMR1* and *VAN1* in immune evasion.

## DISCUSSION

Neutrophils are critical for the host response to *Candida* (8, 9). However, neutrophils are less effective at engaging, phagocytosing, and killing C. auris (14). The current studies show how C. auris cell wall mannosylation influences neutrophil interactions, promoting neutrophil evasion for this species. Genetic disruptions of mannosylation pathways allow for increased phagocytosis and killing by human neutrophils. Furthermore, we find that C. auris mannosylation affects neutrophil recruitment and phagocytosis in zebrafish. Similar to studies with human neutrophils, zebrafish neutrophils fail to recruit and kill wild-type C. auris. In contrast, disruption of mannosylation in the *van1*Δ and *pmr1*Δ mutants allows for neutrophil recruitment and phagocytosis *in vivo*. Disruption of this outer cell wall mannan layer led to effective control of C. auris burden in the zebrafish model. Using transgenic zebrafish, we found that control of the infection was due to the activity of neutrophils against the mannan-deficient C. auris strains.

The cell wall of C. auris effectively masks PAMPs, including β-glucan and chitin (Fig. 4) (38). We found C. auris *PMR1* and *VAN1* to contribute to production of the outer cell wall mannan that conceals these immunostimulatory moieties. With unmasking of the glucan in *pmr1*Δ and *van1*Δ mutants, human neutrophils likely engage the C. auris β-glucan via CR3 (CD11b/CD18), as has been elegantly described for glucan particles and C. albicans (39, 40). C. auris mannosylation appears critical for protection from neutrophil phagocytosis.

Recent studies have begun to shed light on the structure of the C. auris cell wall (25, 41). Compared to mannan from other *Candida* spp., Candida auris mannan is noted to be enriched with β-1,2 linkages, and this alteration is proposed to alter host recognition (25). In work by Bruno and colleagues (41), C. auris mannans were found to contain a unique structure containing two distinct α-1,2-mannose-phosphate (Man-α-1-phosphate) sidechains. This structure was not found in C. albicans and isolated mannans from the two species were shown to exhibit differential affinity for two receptors (dectin-2 and mannose receptor). Our studies did not reveal this C. auris mannan structure. This may represent a strain difference or a structural difference related to different growth conditions. Given that our study utilized that same method for mannan isolation and analysis, we do not suspect the absence of the α-1,2-mannose-phosphate (Man-α-1-phosphate) sidechains was related to a difference in protocol.

Previous study of C. auris interaction with both human and murine neutrophils has described a range of effectiveness in antifungal immune responses (41–46). As seen in Fig. 1, different strains of C. auris can vary in triggering neutrophil phagocytosis. Thus, it is likely that C. auris strain differences contribute to the variation in neutrophil responses reported in the current body of literature. Furthermore, differences in the neutrophil receptors that recognize C. albicans cell wall components have been noted for mice and humans, which is another factor likely contributing to differences observed for C. auris-neutrophil interactions in the literature (39, 47, 48). Future study will be integral for understanding mechanisms involved in the range of innate immune responses to various C. auris isolates.

We were surprised to find that *PMR1* and *VAN1* did not appear to play similar roles in modulating neutrophil engagement for either C. albicans or C. glabrata. As described in these studies and prior work, human neutrophils more rapidly and preferentially engage C. albicans compared to C. auris (14). Therefore, initial engagement of PAMPs or other components likely varies between these species. Based on our observations for the *pmr1*Δ/Δ and *van1*Δ/Δ mutants, disruption of mannosylation does not appear to alter that recognition for C. albicans. Prior work with *pmr1*Δ/Δ found the strain to associate with neutrophils in a manner similar to wild type (49). However, engulfment was impaired by the lack of mannosylation. In other work, disruption of *PMR1* in C. albicans did not impact NET formation or neutrophil killing during planktonic growth (50). Neutrophil engagement of C. glabrata was not as robust as that observed for C. albicans. However, disruption of mannosylation by *PMR1* or *VAN1* similarly did not impact neutrophil responses. Therefore, the role of these mannosylation pathways for C. auris neutrophil evasion appears to be divergent from C. albicans and C. glabrata.

## MATERIALS AND METHODS

### Organisms and inoculum.

C. auris (B11203) was utilized as a reference strain and for the generation of C. auris mutant strains *van1Δ*, *pmr1Δ*, and *van1Δ+VAN1* (6). C. albicans and C. glabrata strains were obtained from prior work (51, 52). Strains utilized in this study are listed in Table S2 in the supplemental material. All strains were maintained on yeast extract peptone dextrose (YPD) plates. Cultures were grown overnight in YPD broth on an orbital shaker at 200 rpm at 30°C. After overnight growth, cultures were diluted 1:15 in fresh YPD broth and grown for 2 h in an orbital shaker at 180 rpm, unless otherwise specified, washed twice in Dulbecco’s phosphate-buffered saline (DPBS), and counted with a hemocytometer.

10.1128/mSphere.00406-21.3 TABLE S2 Strains used in this study. Download Table S2, DOCX file, 0.03 MB. Copyright © 2021 Horton et al. 2021 Horton et al. https://creativecommons.org/licenses/by/4.0/ This content is distributed under the terms of the Creative Commons Attribution 4.0 International license.

### Human neutrophil collection.

Human blood was obtained from volunteering donors with informed written consent through a protocol that was approved by the Internal Review Board of the University of Wisconsin-Madison. Neutrophils were isolated as previously described using MACSxpress negative antibody selection kit and purified with the MACSxpress erythrocyte depletion kit (Miltenyi Biotec, Inc., Auburn, CA) (14). Isolated neutrophils were resuspended in RPMI 1640 (lacking phenol red) supplemented with glutamine (0.3 mg/ml) and 2% heat-inactivated fetal bovine serum (FBS). Incubations involving neutrophils were performed at 37°C with 5% CO_2_.

### Generation of mutants and complement strains.

Generation of mutant strains was accomplished by fusion PCR-based disruption of genes based on the methods from Grahl et al. (19), but without usage of CRISPR-Cas9 RNA-protein complexes. In brief, the nourseothricin resistance cassette (*NAT1*) was amplified from the pNat plasmid (53) and 0.5- to 1-kb regions of the gene of interest were amplified in both the 5′ and 3′ directions using isolated genomic DNA from C. auris (MasterPure yeast DNA purification kit, Lucigen). The subsequent fusion PCR yielded the *NAT1* cassette flanked by the 5′ and 3′ regions of the gene targeted for knockout. Primers utilized in these experiments are listed in Table S3. The purification of PCR products was performed with Wizard SV Gel and PCR Clean-up System (Promega) according to the manufacturer’s instructions. Transformations were performed via electroporation based on methods from Grahl et al. (19) with slight modifications. An overnight culture of C. auris in 30 ml of YPD was adjusted to an optical density at 600 nm (OD_600_) of 1.6 to 2.2, pelleted by centrifugation, and resuspended in 20 ml of transformation buffer, containing 10 mM Tris-HCl, 1 mM EDTA, and 100 mM LiAc in ddH_2_O. Cells were then placed on a MACSmix tube rotator (Miltenyi Biotec, Inc., Auburn, CA) at 9 rpm. Following 1 h of incubation at room temperature, 100 mM dithiothreitol (DTT) was added before a subsequent 30-min incubation. Cells were then washed twice in ice-cold ddH_2_O, once in ice-cold 1 M sorbitol, and resuspended in 200 μl of ice-cold 1 M sorbitol. This cell suspension (40 μl) was combined with 1 μg of *NAT1* cassette for electroporation with a manual 1.8 kV pulse (Gene Pulser II, Bio-Rad). Cells were then immediately resuspended in ice-cold 1 M sorbitol, centrifuged at 840 rcf for 3 min, and resuspended in 1 ml YPD. Following 2 h of incubation at 30°C with shaking, cells were plated on YPD plates containing 200 μg/ml nourseothricin and incubated at 30°C. Individual colonies were plated on fresh YPD plates containing 200 μg/ml nourseothricin before genotypic and phenotypic characterization. Complementation of mutants was performed using hygromycin B resistance, utilizing the pYM70 plasmid (54) and generating a cassette with the *HYG* resistance gene at the tail end of the PCR product (52). Despite multiple attempts, we were unable to construct a complemented strain for *pmr1Δ*. Transformation and selection of complements were performed as described above. Colonies were selected on YPD plates containing 200 μg/ml nourseothricin and 200 μg/ml hygromycin B.

10.1128/mSphere.00406-21.4 TABLE S3 Primers used to construct C. auris mutants. Download Table S3, DOCX file, 0.02 MB. Copyright © 2021 Horton et al. 2021 Horton et al. https://creativecommons.org/licenses/by/4.0/ This content is distributed under the terms of the Creative Commons Attribution 4.0 International license.

### Phagocytosis assays.

*Candida* strains were stained with calcofluor white (100 μg/ml) in the dark for 10 min at room temperature, washed three times in DPBS, and added at 4 × 10^6^ cells to a tissue-cultured microslide (Ibidi). Staining with calcofluor white had no effect on viability of the C. auris wild type (WT) or the *pmr1*Δ and *van1*Δ strains, with stained yeast growing to 101.8 ± 9.5, 110.8 ± 24.8, and 95.5 ± 13.5% of unstained controls, respectively. Neutrophils were fluorescently labeled with calcein acetoxymethyl (AM) at 0.5 μg/ml (Thermo Fisher Scientific, Waltham, MA) in the dark for 10 min at room temperature and 1 × 10^6^ cells were added to the microslide wells. After 1 h of incubation, images were obtained with a Nikon eclipse-TI2 inverted microscope equipped with an ORCA-Flash 4.0 LT sCMOS camera, TI2-S-SS-E motorized stage, stage top TIZW series Neco incubation system (Tokai Hit), and NIS elements imaging software with DAPI (4′,6-diamidino-2-phenylindole) (Ex 378/52:Em 447/60 nm) and fluorescein isothiocyanate (FITC) (Ex 466/40: EM 525/50) filters. Subsequently, neutrophils engaged in phagocytosis of C. auris yeast were enumerated and the percentage of engaged neutrophils was calculated by the formula (engaged neutrophils in frame/total neutrophils in frame) × 100, as described previously (14).

### Killing assays.

For killing assays, we utilized PrestoBlue staining of viable C. auris following coculture with neutrophils and lysis of neutrophils (55). C. auris yeast (2 × 10^6^ cells) and neutrophils (1 × 10^6^ cells) were added to wells of a black 96-well microtiter flat-bottom plate (Corning) in triplicate and incubated for 4 h. Wells containing yeast alone and neutrophils alone were included as controls. After incubation, 10 μl of 10 mg/ml DNase I was added to each well to cleave any extracellular DNA and the plate was incubated for 20 min at 37°C with 5% CO_2_. Contents of the wells were removed and placed into a 96-well U-bottom plate (allowing for efficient plate centrifugation). Because some neutrophils and yeast can adhere to the flat-bottom plate, we processed the contents of both the U-bottom and flat-bottom plates and ultimately combined them back in the flat-bottom plates for analysis. Following centrifugation of the U-bottom plate (1,200 × *g*), 100 μl of a 100 μg/ml DNase I solution in ddH_2_O was added with pipette mixing and the plate was incubated 20 min to lyse neutrophils. The residual adherent cells in the flat-bottom plates were similarly treated. After this 20 min incubation, the U-bottom plate was centrifuged, and supernatant was discarded. Then, the contents of the flat-bottom plate were removed and used to resuspend the pellets in the U-bottom plate, ensuring to keep the same well orientation between plates. Fresh DNase I solution was added to the flat-bottom plate, and both plates were incubated for 20 min, then this process was repeated to ensure lysis of any remaining neutrophils. The flat-bottom plate was left empty at room temperature during the final 20 min incubation of the U-bottom plate. After this final incubation, supernatant was removed from the U-bottom plate, 90 μl of DPBS was added to the wells of the U-bottom plate with vigorous pipetting, and the contents were transferred to the corresponding wells in the flat-bottom plate. Then, a 1:10 dilution of PrestoBlue reagent was made in RPMI + 2% FBS and 110 μl of this solution was added to the wells of the U-bottom plate with vigorous pipette mixing. The contents were again transferred to corresponding wells in the flat-bottom plate containing yeast without viable neutrophils. The flat-bottom plate was incubated for 2 h at 37°C with 5% CO_2_ before reading fluorescence at 560/590 nm in a microplate reader (Synergy H1, Bio-Tek Instruments). The percentage of viable yeast was quantified by calculating fluorescence signal from yeast incubated with neutrophils as a percentage of the same strain incubated without neutrophils. The fluorescence levels of neutrophil-only controls were subtracted from the values of wells containing neutrophils and yeast.

### Scanning electron microscopy.

Interactions of C. auris strains and neutrophils were observed with scanning electron microscopy as previously described (14, 50). Briefly, individual strains of C. auris yeast were incubated with neutrophils for 1 h. Subsequently, samples were rinsed with DPBS and fixed overnight in a solution of 4% formaldehyde, 1% glutaraldehyde in PBS. Samples were then washed with PBS, treated with 1% osmium tetroxide, and washed again in PBS. Afterward, samples were subjected to dehydration through a series of ethanol washes before critical point drying and mounting on aluminum stubs. Samples were sputter coated with platinum and imaged with a scanning electron microscope (LEO 1530) at 3 kV.

### Susceptibility to oxidative stress and cell wall perturbation.

Overnight cultures were enumerated with a hemocytometer and adjusted to 5 × 10^4^ cells/ml in RPMI 1640 (Corning). Then, 100 μl of yeast inoculum was added in duplicate to a clear round-bottom microtiter plate (Falcon). Dilution series of H_2_O_2_ (128 to 0.25 mM), menadione (512 to 1μM), Congo red (2 to 0.00390625 μg/ml), and calcofluor white (256 to 0.5 μg/ml) were prepared in RPMI 1640 and added to wells. The lid of the microtiter plates was wrapped in Parafilm and incubated at 37°C with 5% CO_2_ for 24 h before optically determining the MIC visually.

### Transmission electron microscopy.

Transmission electron microscopy was utilized to analyze the cell walls of C. auris strains, as described previously (56). Briefly, cells were fixed in 4% formaldehyde and 2% glutaraldehyde, postfixed with 1% osmium tetroxide and 1% potassium ferricyanide, stained with 1% uranyl acetate, dehydrated through a graded series of ethanol solutions, and embedded in Spurr’s resin. Sections (70 nm) were cut and placed on copper grids, poststained with 8% uranyl acetate in 50% methanol and Reynolds’ lead citrate, and analyzed with a transmission electron microscope (Philips CM 120). Cell wall lengths were manually measured using Fiji.

### Cell wall monosaccharide analysis.

Overnight cultures of C. auris strains were rinsed twice in DPBS, counted with a hemocytometer, and adjusted to 1 ml of 5 × 10^7^ cells/ml in DPBS in 2-ml micro-tubes (Sarstedt) containing glass beads. Cells were placed in a bead beater (Mini-Beadbeater, Biospec Products) at full speed for 1-min increments for a total of 5 min, with 1-min incubations on ice in between bead-beating repeats. The contents of tubes (except for glass beads) were transferred to fresh 1.5-ml Eppendorf tubes, then rinsed six times with ddH_2_O with centrifugation at 1,200 × *g* to pellet cell walls. After drying overnight, cell walls were hydrolyzed in 2 N trifluoroacetic acid (Sigma-Aldrich) at 120°C for 90 min. Carbohydrates were analyzed based on the modified procedures (57). Monosugars were converted to alditol acetate derivatives (58) and then identified and quantified by gas chromatography on a Shimadzu GC-2010 system (Shimadzu). A Crossbond 50% cyanopropylmethyl/50% phenylmethyl polysiloxane column was used (15 m × 0.25 mm with 0.25 μm film thickness, RTX-225, Restek). The analysis conditions included: injector at 220°C, FID detector at 240°C, and a temperature program of 215°C for 2 min, then 4°C/min up to 230°C before holding for 11.25 min, run at constant linear velocity of 33.4 cm/sec and split ratio of 50:1.

### Mannan isolation.

Frozen samples were thawed and 1 ml of paraformaldehyde was added to the cell slurry. Cells were incubated at room temperature for 1 h, washed 3 times with H_2_O, and the cells were added to 20 × 125 mm screw cap, round bottom Kimax tubes (Kimble). Briefly, 10 ml of 0.75 N NaOH was added to the packed cells in each tube, followed by vortexing to produce a suspension. All samples were extracted in parallel in a heating mantle with adapters that accommodate 20 × 125 mm tubes. The heating mantle was preheated to 60°C and the glass tubes were equilibrated at that temperature, followed by temperature ramping to 100°C. The yeast suspensions were incubated for 15 min at 100°C with intermittent shaking to prevent settling. The total heating time was ∼30 min. The tubes were removed from the heating mantle and allowed to cool. The tubes were centrifuged for 10 min at 863 × *g*, until the cell stroma formed a pellet. The supernatant containing mannan was decanted and added to a 30-ml capacity 2000 MWCO Slide-A-Lyzer (Thermo Scientific). The mannan solutions were dialyzed against 400 volumes of 18 megohm Type I water. The pH of the final product was ∼7.0. The solutions were frozen, lyophilized, and stored at −20°C in a dry environment until analyzed. Yields ranged between 8.5 and 26 mg.

### NMR analysis.

Nuclear magnetic resonance (NMR) data acquisition and analysis are based on methods described by Lowman et al. (59). In summary, ^1^H NMR spectra for mannan were collected on a Bruker Avance III 400 NMR spectrometer operating at 333°K (60°C) in 5-mm NMR tubes. Mannan (about 10 mg) was dissolved in about 600 μl D_2_O (Cambridge Isotope Laboratories, 99.8+% deuterated). Chemical shift referencing was accomplished relative to trimethylsilylpropionate (TMSP) at 0.0 ppm. NMR spectra were collected and processed as follows: 200 scans, 65,536 data points, 20.69 ppm sweep width centered at 6.175 ppm, and 1 s pulse delay. Spectra were processed using exponential apodization with 0.3 Hz line broadening. COSY spectra were collected as 2048 by 128, processed as 1024 by 1024, 16 prescans, 100 scans, sweep width 10 ppm centered at 4.5 ppm, and relaxation delay 1.49 sec. Processing was accomplished with sine apodization in both dimensions using TopSpin (version 4.0.9) on the MacBook Pro. To determine the relative changes in structural motifs, spectra were overlaid with the resonance at the α1→6-linked mannosyl repeat units (5.07 ppm).

### Fluorescent labeling of cell wall components.

The amounts of exposed β-glucan on the cell wall surface were quantified using methods from Nogueria et al. (30), with modifications. Briefly, *Candida* cells were adjusted to 1 × 10^7^ cells/ml and suspended in 1 ml of 4°C blocking solution (composed of 0.5% bovine serum albumin [BSA], 5% HI-fetal bovine serum, 5 mM EDTA, and 2 mM NaN_3_ in 1× DPBS) and incubated for 30 min at room temperature on a MACSmix tube rotator (Miltenyi Biotec, Inc., Auburn, CA) at 9 rpm. Cells were collected via centrifugation and washed twice with cold flow cytometry washing solution (0.5% BSA, 5 mM EDTA, and 2 mM NaN_3_ in DPBS) and then labeled with Fc: dectin-1 protein (Fc [human]: dectin-1 [mouse] [recombinant, Adipogen]) at 1 μg/ml in blocking solution for 1 h on ice. Blocking buffer-only controls were included for each strain. Cells were subsequently washed three times with washing solution, and resuspended in a 1:200 dilution of Alexa Fluor 488-conjugated anti-human IgG Fc antibody (Fc + 488; Biolegend) in blocking solution. Cells were incubated on ice in the dark for 45 min, washed three times with washing solution, and resuspended to 1 × 10^7^ cells/ml in washing solution. Cell suspension (150 μl) was added in triplicate to wells of a black 96-well microtiter plate (Costar) and fluorescence intensity was quantified in a microplate reader at 488/519 nm. Chitin exposure was similarly analyzed, using wheat germ agglutinin conjugated to fluorescein isothiocyanate (WGA-FITC) to label exposed chitin (31). Briefly, *Candida* cells were adjusted to 1 × 10^7^ cells/ml and suspended in 1 ml of 4°C DPBS containing 2% BSA (wt/vol). Cells were incubated for 30 min at room temperature on a MACSmix tube rotator (Miltenyi Biotec, Inc., Auburn, CA) at 9 rpm. Cells were collected via centrifugation and washed twice with cold DPBS containing 0.05% Tween 20 (vol/vol) and then labeled with 0.1 mg/ml WGA-FITC in tubes containing DPBS with 1% BSA (wt/vol). Unstained controls were included for each strain. Cells were incubated at room temperature in the dark for 1 h. Cells were washed three times with DPBS + 0.05% Tween 20 and resuspended to 1 × 10^7^ cells/ml in DPBS + 0.05% Tween 20. Cell suspension (150 μl) was added in triplicate to wells of a black 96-well microtiter plate (Costar) and fluorescence intensity was quantified in a microplate reader at 488/519 nm.

Fluorescent microscopy of the β-glucan-labeled and the chitin-labeled yeast was performed. Briefly, the fluorescently labeled yeast were added to a well of a microslide (Ibidi) and imaged on a Nikon eclipse-TI2 inverted microscope equipped with an ORCA-Flash 4.0 LT sCMOS camera, TI2-S-SS-E motorized stage, stage top TIZW series Neco incubation system (Tokai Hit), and NIS elements imaging software with the FITC filter at 30× magnification.

### Zebrafish maintenance.

All animal procedures were approved by the Institutional Animal Care and Use Committee at the University of Wisconsin according to the guidelines of the Animal Welfare Act and The Institute of Laboratory Animal Resources Guide for the Care and Use of Laboratory Animals. Adult zebrafish and larvae were maintained as previously described (32, 33). Larvae were manually dechorionated at 1 day postfertilization (dpf).

### Zebrafish fungal burden.

Hindbrain injections of C. auris were performed as described (14, 32), with an inoculum of 5 × 10^7^ yeast/ml in DPBS in wild-type zebrafish (Danio rerio) or transgenic fish lacking functional neutrophils (Tg[mpx:mCherry-2A-Rac2D57N]) (D57N) (37). Phenol red (1%) was mixed with a 7.5 × 10^7^ yeast/ml suspension in a 1:2 ratio to easily visualize the inoculum in the larval hindbrain after injection and to achieve a concentration of 5 × 10^7^ yeast/ml. Two doses of 3-nl infection doses were successively injected in the hindbrains of larvae at day 0. Injected larvae were maintained at 28.5°C in E3-MB for the remainder of the experiment. For fungal burden quantification, zebrafish larvae were placed in individual 1.5-ml microcentrifuge tubes containing 95 μl of 1× DPBS with 500 μg/ml of kanamycin and 500 μg/ml of gentamicin, and homogenized using a mini bead beater (15 s), plated on YPD plates containing 25 μg/ml chloramphenicol, and incubated at 30°C for CFU determination.

### Zebrafish hindbrain imaging of neutrophils.

For quantification of neutrophil recruitment, C. auris at a concentration of 5 × 10^7^ yeast/ml in DPBS was injected into the hindbrains of double-transgenic zebrafish larvae *Tg*(*lyzc:tagRFP*) × *Tg*(*mpeg:EGFP*). Prior to imaging experiments, larvae were maintained in E3 without methylene blue (E3-MB) containing 0.2 mM N-phenyl thiourea (PTU, Sigma-Aldrich) beginning at 1 day postfertilization (dpf). Injected larvae were maintained at 28.5°C in E3-MB containing PTU for the remainder of the experiment. Hindbrains of injected larvae were imaged according to methods from Schoen et al. (33). Larvae were screened for the presence of fluorescence markers with a zoomscope (EMS3/SyCoP3; Zeiss; Plan-NeoFluar Z objective). A spinning disc confocal microscope (CSU-X; Yokogawa) with a confocal scanhead on a Zeiss Observer Z.1 inverted microscope, Plan-Apochromat NA 0.8/20x objective, and a Photometrics Evolve EMCCD camera were used for multiday imaging experiments. Larvae that were imaged at multiple time points were kept in a 24-well plate in E3-MB with PTU. At imaging time points, larvae were anesthetized using E3-MB containing tricaine, then placed into a zWEDGI chamber (60). All z-series images were acquired in 5-μm slices and larvae were oriented for full visibility of the hindbrain. Once imaging was performed, larvae were rinsed with E3-MB and placed back into the 24-well plate containing E3-MB with PTU. Images were acquired with ZEN software (Zeiss). Zebrafish hindbrain images were generated as maximum intensity projections of z-series images in Fiji. Neutrophil and macrophage recruitment was quantified by manual counts within the region of interest (ROI), which was manually defined from the brightfield image to encompass the hindbrain. In addition, macrophage recruitment was quantified through manually thresholding the green fluorescence protein (GFP) from macrophages in the ROI and measuring the area of GFP signal therein. Quantification of both counts and area of GFP signal were performed from maximum intensity projections from z-stacks in Fiji (33).

### Statistics.

Experiments were performed at least 3 times using neutrophils from different donors on different days. Zebrafish studies included at least nine animals per condition. Statistical analyses were performed by one-way or Brown-Forsythe and Welch ANOVA with Holm-Sidak pairwise comparisons or Student’s *t* test using GraphPad Prism software. Differences of *P* < 0.05 were considered significant.

10.1128/mSphere.00406-21.1 TEXT S1 Supplemental methods.Text S1, DOCX file, 0.02 MB Copyright © 2021 Horton et al. 2021 Horton et al. https://creativecommons.org/licenses/by/4.0/ This content is distributed under the terms of the Creative Commons Attribution 4.0 International license.

10.1128/mSphere.00406-21.5 TABLE S4 Primers utilized for quantitative PCR. Download Table S4, DOCX file, 0.02 MB. Copyright © 2021 Horton et al. 2021 Horton et al. https://creativecommons.org/licenses/by/4.0/ This content is distributed under the terms of the Creative Commons Attribution 4.0 International license.
