# Supplementary figures and images for: Candida auris Cell Wall Mannosylation Contributes to Neutrophil Evasion through Pathways Divergent from Candida albicans and Candida glabrata
Source: mSphere. 2021 Jun 23;6(3):e00406-21. doi: 10.1128/mSphere.00406-21 (PMC8265655; doi:10.1128/mSphere.00406-21)

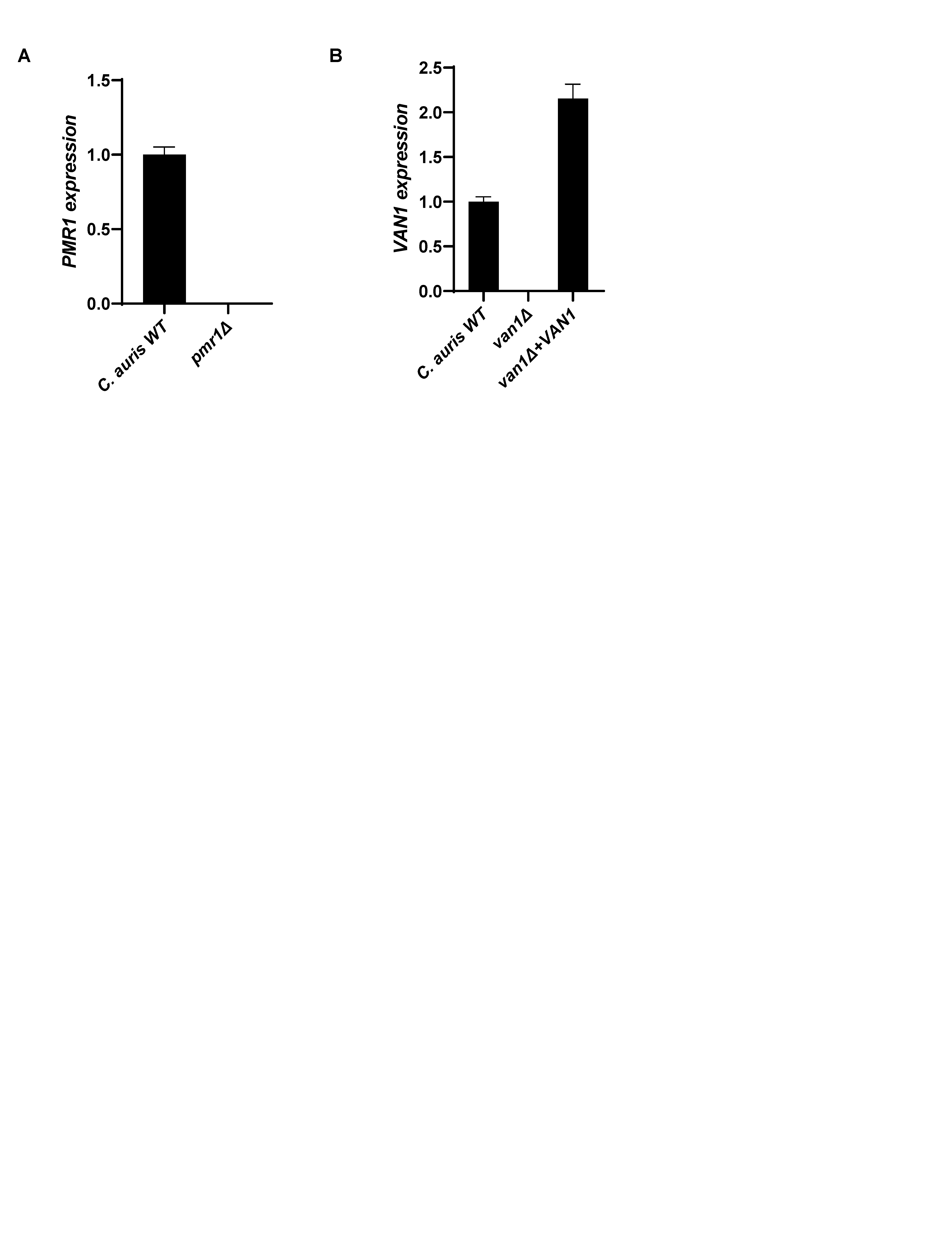

Supplement: FIG S1 [file msphere.00406-21-sf001.tif]

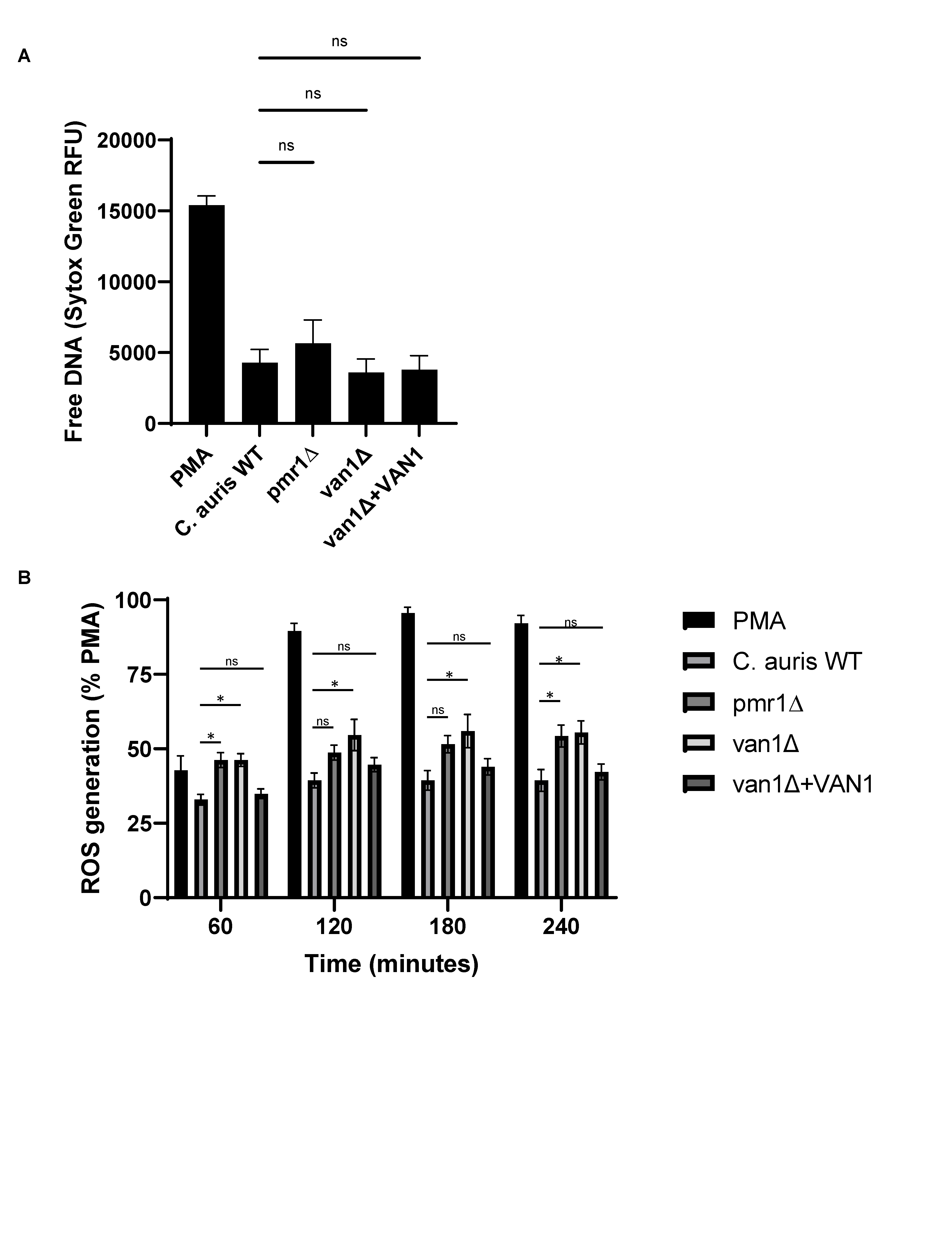

Supplement: FIG S2 [file msphere.00406-21-sf002.tif]

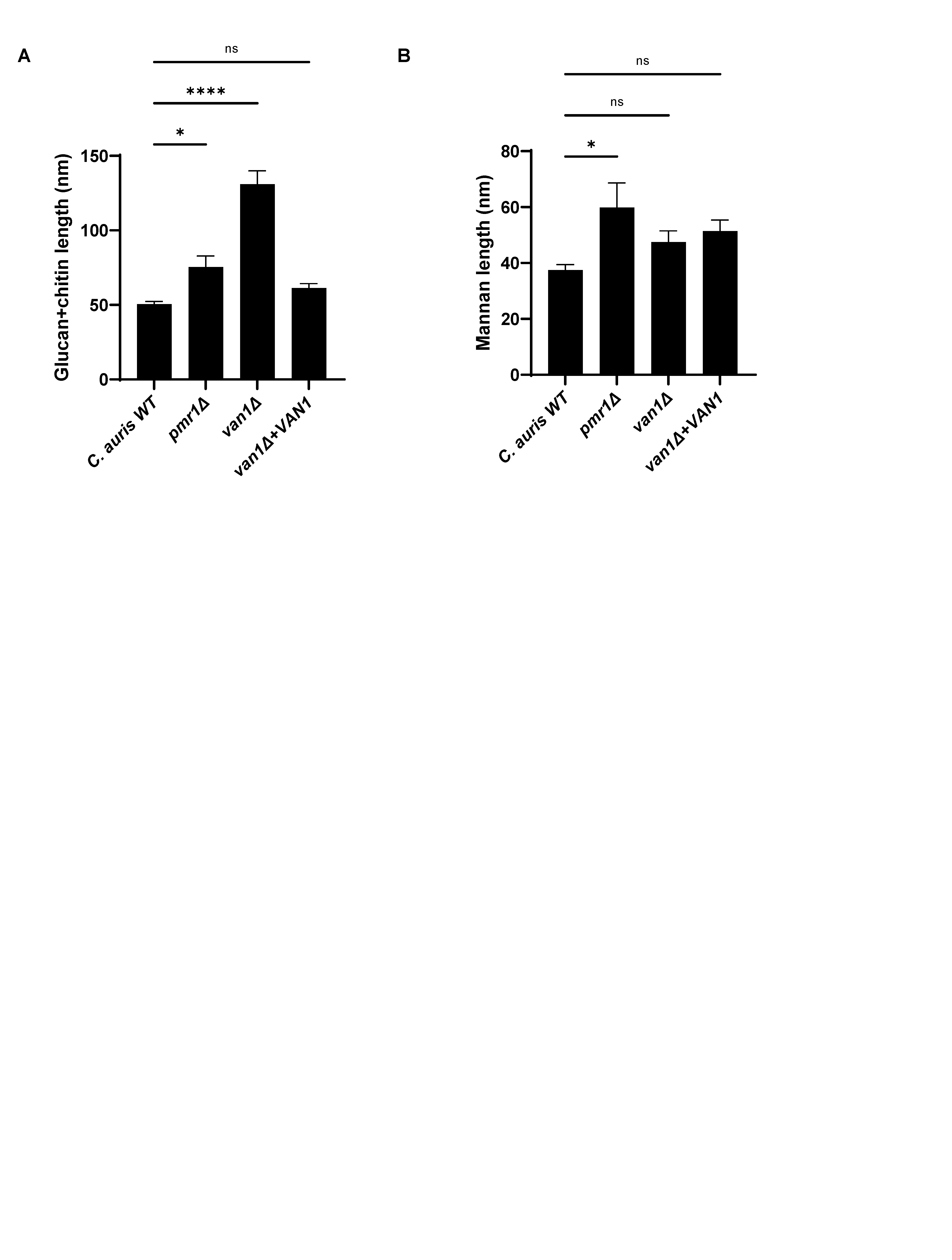

Supplement: FIG S3 [file msphere.00406-21-sf003.tif]

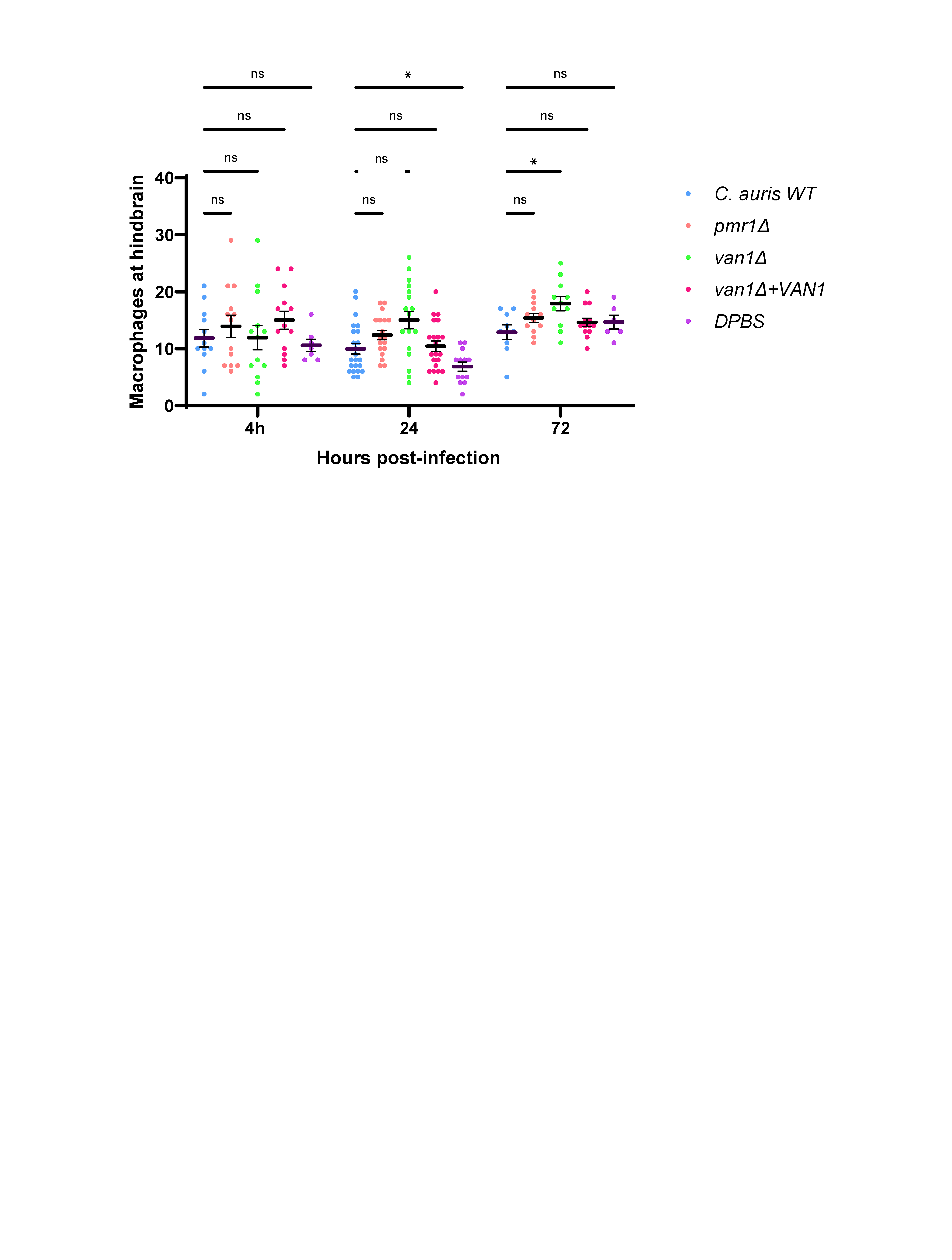

Supplement: FIG S4 [file msphere.00406-21-sf004.tif]

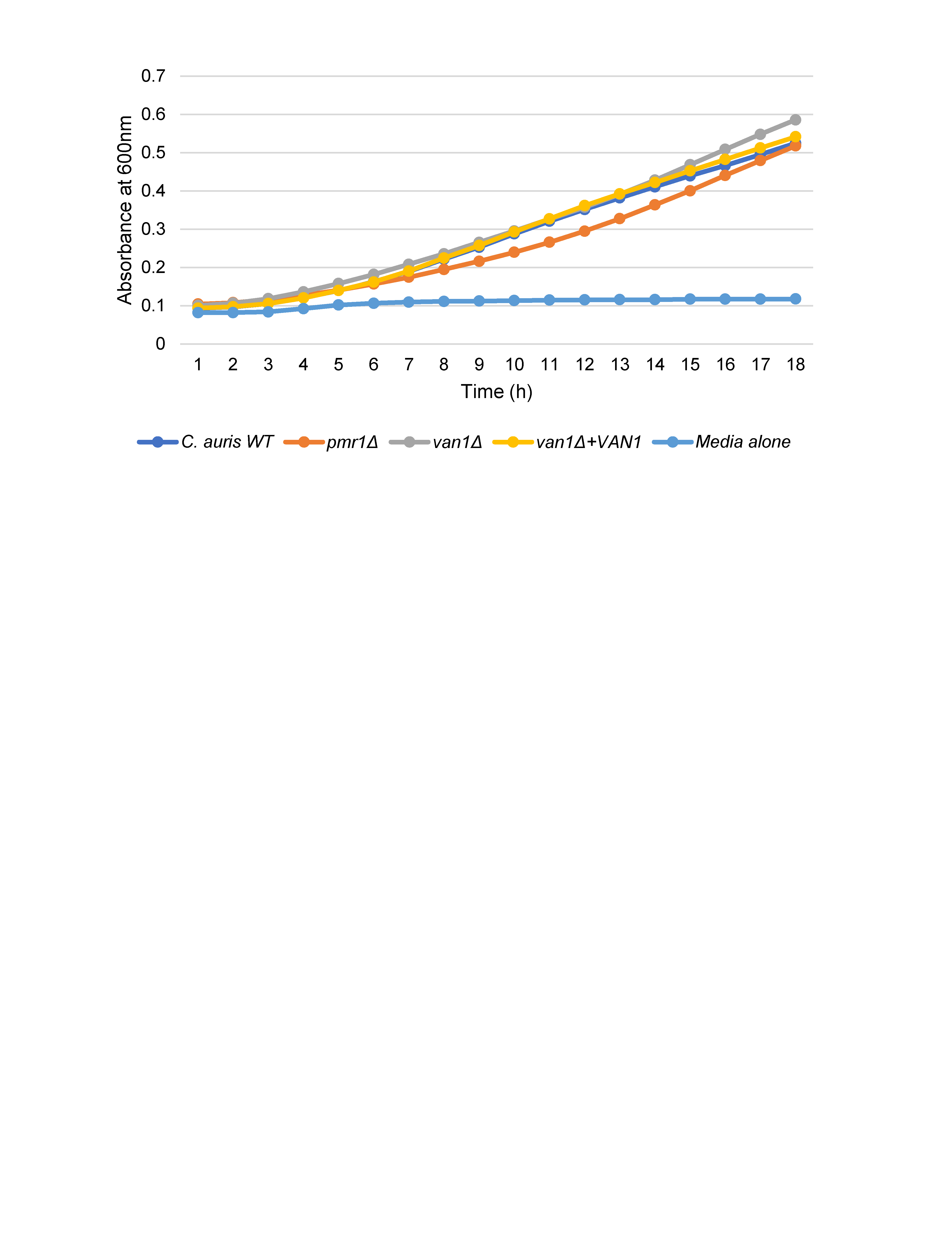

Supplement: FIG S5 [file msphere.00406-21-sf005.tif]
