# Supplementary material for: Candida auris Cell Wall Mannosylation Contributes to Neutrophil Evasion through Pathways Divergent from Candida albicans and Candida glabrata
Source: mSphere. 2021 Jun 23;6(3):e00406-21. doi: 10.1128/mSphere.00406-21 (PMC8265655; doi:10.1128/mSphere.00406-21)
Supplement: TABLE S1 [file msphere.00406-21-st001.docx]

**Table S1: Minimal inhibitory concentration values of cellular stressors**

| **MIC values** | Hydrogen peroxide (µM) | Menadione (µM) | Calcoflour White (µg/mL) | Congo Red (µg/mL) |
| --- | --- | --- | --- | --- |
| *C. auris* WT | 16 | 64 | 16 | 2 |
| *pmr1Δ* | 16 | 64 | 4 | 0.5 |
| *van1Δ* | 16 | 64 | 4 | 0.5 |
| *van1Δ+VAN1* | 16 | 64 | 16 | 2 |
