# Supplementary material for: Candida auris Cell Wall Mannosylation Contributes to Neutrophil Evasion through Pathways Divergent from Candida albicans and Candida glabrata
Source: mSphere. 2021 Jun 23;6(3):e00406-21. doi: 10.1128/mSphere.00406-21 (PMC8265655; doi:10.1128/mSphere.00406-21)
Supplement: TABLE S2 [file msphere.00406-21-st002.docx]

**Table S2: Strains used in this study**

| Organism | Gene | Strain | Genotype | Source |
| --- | --- | --- | --- | --- |
| *C. auris* | Reference | B11203 | Clinical isolate (India) | [1], CDC |
| *C. auris* | *pmr1Δ* | MVH3 | *pmr1Δ::NAT1* | This study |
| *C. auris* | *van1Δ* | MVH2 | *van1Δ::NAT1* | This study |
| *C. auris* | *van1Δ*+*VAN1* | MVH4 | *van1Δ::NAT1::VAN1/HYG* | This study |
| *C. auris* | N/A | B11219 | Clinical isolate (India) | [1], CDC |
| *C. auris* | N/A | B11211 | Clinical isolate (India) | [1], CDC |
| *C. auris* | N/A | B11104 | Clinical isolate (Pakistan) | CDC |
| *C. auris* | N/A | B11804 | Clinical isolate (Colombia) | CDC |
| *C. auris* | N/A | B11801 | Clinical isolate (Colombia) | CDC |
| *C. auris* | N/A | B11785 | Clinical isolate (Colombia) | CDC |
| *C. auris* | N/A | B11799 | Clinical isolate (Colombia) | CDC |
| *C. auris* | N/A | B11220 | Clinical isolate (Japan) | [1], CDC |
| *C. auris* | N/A | B11221 | Clinical isolate (South Africa) | [1], CDC |
| *C. albicans* | Reference | SN250 | his1Δ/his1Δ, leu2Δ::C.dubliniensis HIS1 /leu2Δ::C.maltosa LEU2, arg4Δ /arg4Δ, URA3/ura3Δ::imm434, IRO1/iro1Δ::imm434 | [2] |
| *C. albicans* | *pmr1Δ/Δ* | KMR354 | *URA3 IRO1 arg4 his1 leu2 pmr1::C.d HIS1*  *ura3::λimm434 iro1:: λimm434 arg4 his1 leu2 pmr1::C.m LEU2* | [3] |
| *C. albicans* | *van1Δ/Δ* | ELR111 | *URA3 IRO1 arg4 his1 leu2 van1::C.d HIS1*  *ura3::λimm434 iro1:: λimm434 arg4 his1 leu2 van1::C.m LEU2* | [3] |
| *C. glabrata* | Reference | HTL | *his3*∆::FRT, *leu2*∆::FRT, *trp1*∆::FRT | [4] |
| *C. glabrata* | *pmr1Δ/Δ* | EGD143 | *his3*∆::FRT, *leu2*∆::FRT, *trp1*∆::FRT, *pmr1*∆::NAT1 | [5] |
| *C. glabrata* | *van1Δ/Δ* | EGD137 | *his3*∆::FRT, *leu2*∆::FRT, *trp1*∆::FRT, *van1*∆::NAT1 | [5] |

CDC: Centers for Disease Control and Prevention

**References**

1. Lockhart SR, Etienne KA, Vallabhaneni S, Farooqi J, Chowdhary A, Govender NP, et al. Simultaneous Emergence of Multidrug-Resistant Candida auris on 3 Continents Confirmed by Whole-Genome Sequencing and Epidemiological Analyses. Clin Infect Dis. 2017;64(2):134-40. Epub 2016/12/19. doi: 10.1093/cid/ciw691. PubMed PMID: 27988485; PubMed Central PMCID: PMCPMC5215215.

2. Noble SM, French S, Kohn LA, Chen V, Johnson AD. Systematic screens of a *Candida albicans* homozygous deletion library decouple morphogenetic switching and pathogenicity. Nature genetics. 2010;42(7):590-8. doi: 10.1038/ng.605. PubMed PMID: 20543849; PubMed Central PMCID: PMCPMC2893244.

3. Mitchell KF, Zarnowski R, Sanchez H, Edward JA, Reinicke EL, Nett JE, et al. Community participation in biofilm matrix assembly and function. Proceedings of the National Academy of Sciences of the United States of America. 2015;112(13):4092-7. doi: 10.1073/pnas.1421437112. PubMed PMID: 25770218; PubMed Central PMCID: PMC4386410.

4. Schwarzmuller T, Ma B, Hiller E, Istel F, Tscherner M, Brunke S, et al. Systematic phenotyping of a large-scale *Candida glabrata* deletion collection reveals novel antifungal tolerance genes. PLoS pathogens. 2014;10(6):e1004211. doi: 10.1371/journal.ppat.1004211. PubMed PMID: 24945925; PubMed Central PMCID: PMCPMC4063973.

5. Dominguez E, Zarnowski R, Sanchez H, Covelli AS, Westler WM, Azadi P, et al. Conservation and divergence in the *Candida* species biofilm matrix mannan-glucan complex structure, function, and genetic control. mBio. 2018;9(2). doi: 10.1128/mBio.00451-18. PubMed PMID: 29615504; PubMed Central PMCID: PMCPMC5885036.
