# Supplementary material for: Candida auris Cell Wall Mannosylation Contributes to Neutrophil Evasion through Pathways Divergent from Candida albicans and Candida glabrata
Source: mSphere. 2021 Jun 23;6(3):e00406-21. doi: 10.1128/mSphere.00406-21 (PMC8265655; doi:10.1128/mSphere.00406-21)
Supplement: TABLE S3 [file msphere.00406-21-st003.docx]

**Table S3: Primers used to construct *C. auris* mutants**

| **Primer type** | **Name** | **Gene** | **Sequence** |
| --- | --- | --- | --- |
| 1 | Left flank fw | *C. auris PMR1* | CAATTTTCAGGCTGCACAGA |
| 2 | Left flank rev | *C. auris PMR1* | **AACGTCGTGACTGGGAAAaaTCATTA**ATAGGGACCCAGGTGGAATC |
| 3 | Right flank fw | *C. auris PMR1* | **TGTGAAATTGTTATCCGCTCACAATTCCAC**CCTCAAGCTTGCGATAAAGC |
| 4 | Right flank rev | *C. auris PMR1* | ACTAAAGAGTGCGCCCAAAA |
| 7 | Nested fw | *C. auris PMR1* | CTCTGAACGCAACGGAATTT |
| 8 | Nested rev | *C. auris PMR1* | CACATGACGCTCCGACTAGA |
| ICF | Internal check forward | *C. auris PMR1* | TCAGTTGGATCGCTTGTCTG |
| ICR | Internal check reverse | *C. auris PMR1* | CAGCTTGGAAGAAAGGAACG |
| 1 | Left flank fw | *C. auris VAN1* | GGCTCCATGGCAGACAAGTT |
| 2 | Left flank rev | *C. auris VAN1* | **AACGTCGTGACTGGGAAAaaTCATTA**GCGGCAACTAAACGGTTTCC |
| 3 | Right flank fw | *C. auris VAN1* | **TGTGAAATTGTTATCCGCTCACAATTCCAC**GCCCAATTTTGCAGCCAGAA |
| 4 | Right flank rev | *C. auris VAN1* | TTTGGCTGTTGGAAGCGTTG |
| 7 | Nested fw | *C. auris VAN1* | TCGACGACAAGGATGGCATT |
| 8 | Nested rev | *C. auris VAN1* | AGCTATGCGCAAGGTCTGTC |
| ICF | Internal check forward | *C. auris VAN1* | TTTCCGACTGTTCTCCTGGC |
| ICR | Internal check reverse | *C. auris VAN1* | TGCCATCTTTCCGAATGCCT |
| NAT1 fw5 | pNAT forward | *NAT1* | TAATGAttTTTCCCAGTCACGACGTT |
| NAT1 rev6 | pNAT reverse | *NAT1* | GTGGAATTGTGAGCGGATA |
| NAT1 fw9 | Internal check forward | *NAT1* | GAAGTTCCAGTTGATCCACCATTGA |
| NAT1 rev10 | Internal check reverse | *NAT1* | CGATGGTACTGCTTCCGATGG |
| Complement c2 | Complement rev | *C. auris VAN1* | **CACGGCGCGCCTAGCAGCGG**AGGCTTTCTGGCTGCAAAAT |
| Complement c3 | Complement fw | *C. auris VAN1* | **GTCAGCGGCCGCATCCCTGC**TTCAATTGCCAAAGCACCCC |
| Complement nested reverse | Nested reverse | *C. auris VAN1* | ATTGGTTTCGGGGGCTCAAT |
| *HygB* cassette amplification | pYM70 forward | *HygB* | ccgctgctaggcgcgccgtggtatagtgcttgctgttcgat |
| *HygB* cassette amplification | pYM70 reverse | *HygB* | gcagggatgcggccgctgacattttatgatggaatgaatgg |

Note: sequences in bold represent sequences present in the *NAT1* cassette
