# Supplementary material for: Candida auris Cell Wall Mannosylation Contributes to Neutrophil Evasion through Pathways Divergent from Candida albicans and Candida glabrata
Source: mSphere. 2021 Jun 23;6(3):e00406-21. doi: 10.1128/mSphere.00406-21 (PMC8265655; doi:10.1128/mSphere.00406-21)
Supplement: TEXT S1 [file msphere.00406-21-t0001.docx]

**Supplemental Methods**

**RNA isolation and quantitative RT-PCR.** RNA was isolated from planktonic yeast using the RNEasy Protect Mini Kit (Quiagen) and quantified using a NanoDrop spectrophotmeter. For the RT-PCR reaction, the QuantiTect Probe RT-PCR kit (Quiagen) was utilized with a CFX96 real-time PCR detection system (Bio-Rad) using the following program: 50°C for 30 min, initial denaturation at 95°C for 15 min, and then 40 cycles of 94°C for 15 s and 60°C for 1 min, as previously described [1]. Primers are shown in Table S4. The expression of each gene relative to that of *ACT1* is presented. The quantitative data was analyzed via the ΔΔ*C_T_* method (where *C_T_* is the threshold cycle) [2]. Reactions were performed in triplicate.

**Sytox Green assays**. Sytox Green staining was utilized to measure extracellular DNA as a estimate of NET formation, as previously described. Both neutrophils and *C. auris* yeast were each added to a black 96-well microtiter plate (Corning) at 2 x 10^5^ cells/well as previously described [3]. Briefly, C. *auris* yeast were each added to a black 96-well microtiter plate (Corning) at 2 x 10^5^ cells/well with neutrophils (2 x 10^5^ cells/well) for 4 h. Free DNA was quantified via fluorescence (500/528nm) in a microplate reader following additing Sytox Green reagent, with cells wells containing phorbol myristate acetate (PMA [100 nM]) as a positive control for eliciting free DNA.

**Reactive oxygen species generation assays**. Measurement of reactive oxygen species (ROS) was performed as previously described [3]. In brief, neutrophils were incubated with the fluorescent oxidative stress dye CM-H_2_DCFDA (Life Technologies, Inc., Eugene, OR) in DPBS for 10 min in the dark and then rinsed twice with DPBS. Subsequently, the treated neutrophils (2x10^5^ cells/well plate) were mixed with individual *C. auris* strains (2x10^5^ cells/well) in a black 96-well plate and fluorescence (495/527 nm) measurements were acquired every 30 min for 4 h. In a subset of wells, 100 nM PMA was included as a positive control for comparison. Prior to analyzing the data, background fluorescence was determined and subtracted from corresponding total fluorescence values.

**Growth curves.** Overnight cultures of yeast were rinsed twice in DPBS, counted with a hemocytometer, and adjusted to 2 x 10^6^ cells/mL in RPMI supplemented with 2% FBS. 100 µL of yeast solution was placed into a clear 96-well microtiter plate in triplicate, then grown for 18 h in a microplate reader with shaking at 29°C to mimic temperatures inside the zebrafish hindbrain. OD_600_ measurements were taken every hour.

**References**

1. Nett JE, Brooks EG, Cabezas-Olcoz J, Sanchez H, Zarnowski R, Marchillo K, et al. Rat Indwelling Urinary Catheter Model of Candida albicans Biofilm Infection. Infect Immun. 2014;82(12):4931. doi: 10.1128/IAI.02284-14.

2. Livak KJ, Schmittgen TD. Analysis of relative gene expression data using real-time quantitative PCR and the 2(-Delta Delta C(T)) Method. Methods (San Diego, Calif. 2001;25(4):402-8. PubMed PMID: 11846609.

3. Johnson CJ, Davis JM, Huttenlocher A, Kernien JF, Nett JE. Emerging Fungal Pathogen Candida auris Evades Neutrophil Attack. mBio. 2018;9(4):e01403-18.
