# Supplementary material for: Candida auris Cell Wall Mannosylation Contributes to Neutrophil Evasion through Pathways Divergent from Candida albicans and Candida glabrata
Source: mSphere. 2021 Jun 23;6(3):e00406-21. doi: 10.1128/mSphere.00406-21 (PMC8265655; doi:10.1128/mSphere.00406-21)
Supplement: TABLE S4 [file msphere.00406-21-st004.docx]

**Table S4: Primers utilized for quantitative PCR**

| **Gene** | **Primers** | **Probe** |
| --- | --- | --- |
| *PMR1* | Forward: 5’ – AAAGAGCCGTTGCCCTATC - 3’  Reverse: 5’ – ACAGGATGGTGTGGTCTTTATC – 3’ | 5’ - /56FAM/TCTGAGGGACTTCGAGTGTTAGCT/36-TAMSp/ -3’ |
| *VAN1* | Forward: 5’ – GTGGAAGGTTACGCCGAATA – 3’  Reverse: 5’ – CCACACCATCTAGGTCAAGAAC – 3’ | 5’ - /56-FAM/ATCTCGCAAGTACGCCAAATGCAC/36-TAMSp/ -3’ |
| *ACT1* | Forward: 5’ – CCAGGGTATCATGGTTGGTATG – 3’  Reverse: 5’ – CCGTGCTCAATTGGGTATCT – 3’ | 5’ - /56-FAM/ACCGACATACGAATCCTTCTGGCC/36-TAMSp/ -3’ |
